# Supplementary material for: Accelerating multielectron reduction at CuxO nanograins interfaces with controlled local electric field
Source: Nat Commun. 2023 Nov 15;14:7383. doi: 10.1038/s41467-023-43303-1 (PMC10651938; doi:10.1038/s41467-023-43303-1)
Supplement: Supplementary file 1 — Supplementary Information [file 41467_2023_43303_MOESM1_ESM.pdf]

Supplementary Information for

Accelerating multielectron reduction at Cu_xO nanograins interfaces with controlled local electric field

Weihua Guo[1,2†], Siwei Zhang[3,†], Junjie Zhang[4], Haoran Wu[5], Yangbo Ma[1], Yun Song[1], Le Cheng[1], Liang Chang[6], Geng Li[1], Yong Liu[1], Guodan Wei[6], Lin Gan[6], Minghui Zhu[5,*], Shibo Xi[7,*], Xue Wang[8], Boris I. Yakobson[4,*], Ben Zhong Tang[3,9*], Ruquan Ye[1,2*]

[1]Department of Chemistry, State Key Laboratory of Marine Pollution, City University of Hong Kong, Hong Kong 999077, China.

[2]City University of Hong Kong Shenzhen Research Institute, Shenzhen, Guangdong 518057, P. R. China.

[3]Department of Chemistry and the Hong Kong Branch of Chinese National Engineering Research Center for Tissue Restoration and Reconstruction, The Hong Kong University of Science and Technology, Hong Kong 999077, China.

[4]Department of Materials Science and Nano Engineering, Rice University, 6100 Main Street, Houston, Texas 77005, USA.

[5]State Key Laboratory of Chemical Engineering, East China University of Science and Technology, Shanghai 200237, China.

[6]Institute of Materials Research, Tsinghua Shenzhen International Graduate School, Tsinghua University, Shenzhen, Guangdong 518055, China.

[7]Institute of Chemical and Engineering Sciences, A*STAR, Singapore 627833, Singapore.

[8]School of Energy and Environment, City University of Hong Kong, Hong Kong 999077, China.

[9]School of Science and Engineering, Shenzhen Institute of Aggregate Science and Technology, The Chinese University of Hong Kong, Shenzhen (CUHK-Shenzhen), Guangdong 518172, China.

[†]These authors contributed equally to this work.

*Corresponding authors.

E-mail: ruquanye@cityu.edu.hk; tangbenz@cuhk.edu.cn; biy@rice.edu; xi_shibo@ices.astar.edu.sg; minghuizhu@ecust.edu.cn.

This PDF file includes:

Supplementary Fig. 1 to 56

Supplementary Note 1

Supplementary Tables 1 to 4

Supplementary References

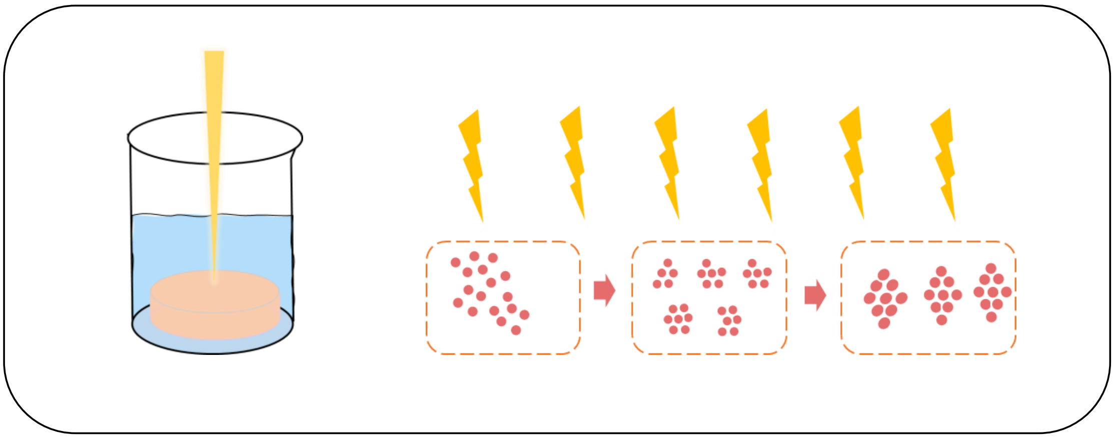

Supplementary Fig. 1. Schematic illustration for the synthesis of L-Cu_xO-HC by laser. Yellow represents laser signal, orange represents copper target, blue represents deionized water, and red represents copper particles.

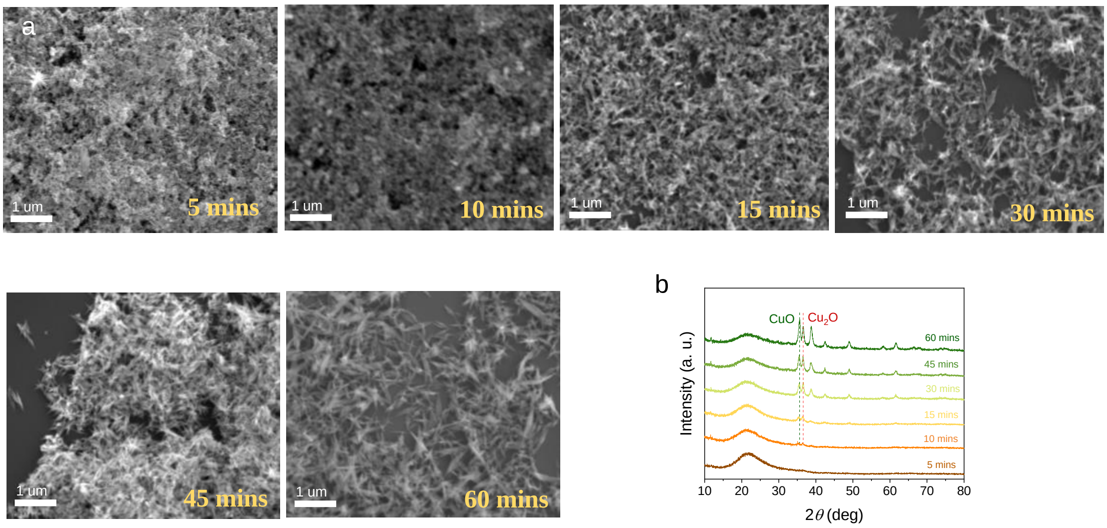

Supplementary Fig. 2. The growth process of L-Cu_xO-HC and its structural evolution during the synthetic process. (a) SEM images of L-Cu_xO-HC in different time during laser ablation. (b) XRD pattern of L-Cu_xO-HC in different. At first, the PLAL procedure yielded dispersed nanoparticles which were characterized as pure amorphous copper oxide (Cu_xO) according to X-ray diffraction (XRD). The formation of oxide nanoparticles could be ascribed to the reaction of Cu nanoparticles generated by laser ablation with dissolved oxygen in water. Afterwards, these were gradually oxidized to Cu_xO nanoparticles (10 mins). Next, the Cu_xO nanoparticles self-assembled into loosely interconnected agglomerates and further merged to form a double vertebral body structure (30 mins), which kept growing and ripening, to develop into CuO bundles eventually (60 mins).

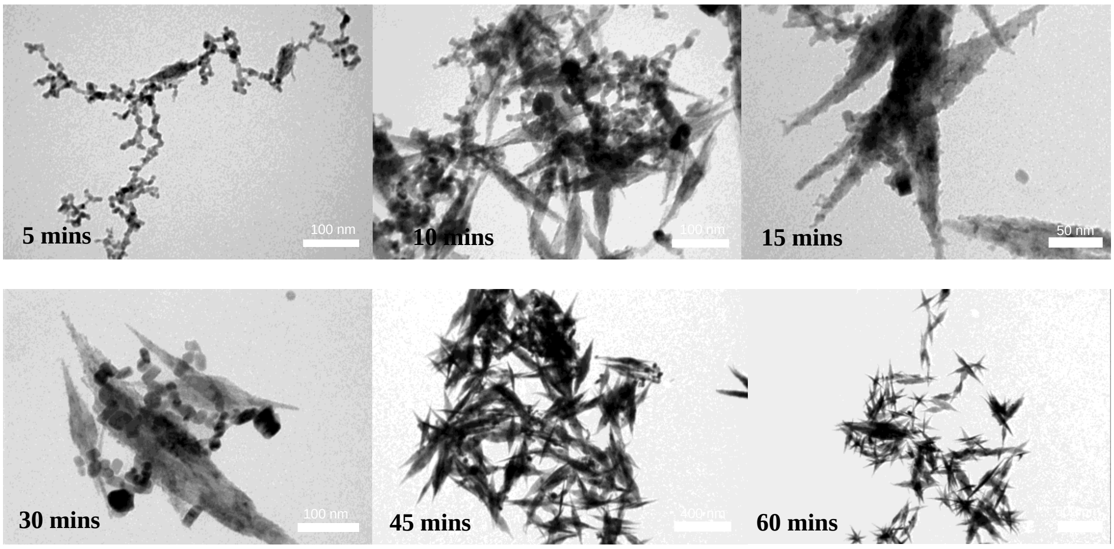

Supplementary Fig. 3 The growth process of L-Cu_xO-HC and its structural evolution during the synthetic process. TEM of L-Cu_xO-HC in different time during laser ablation.

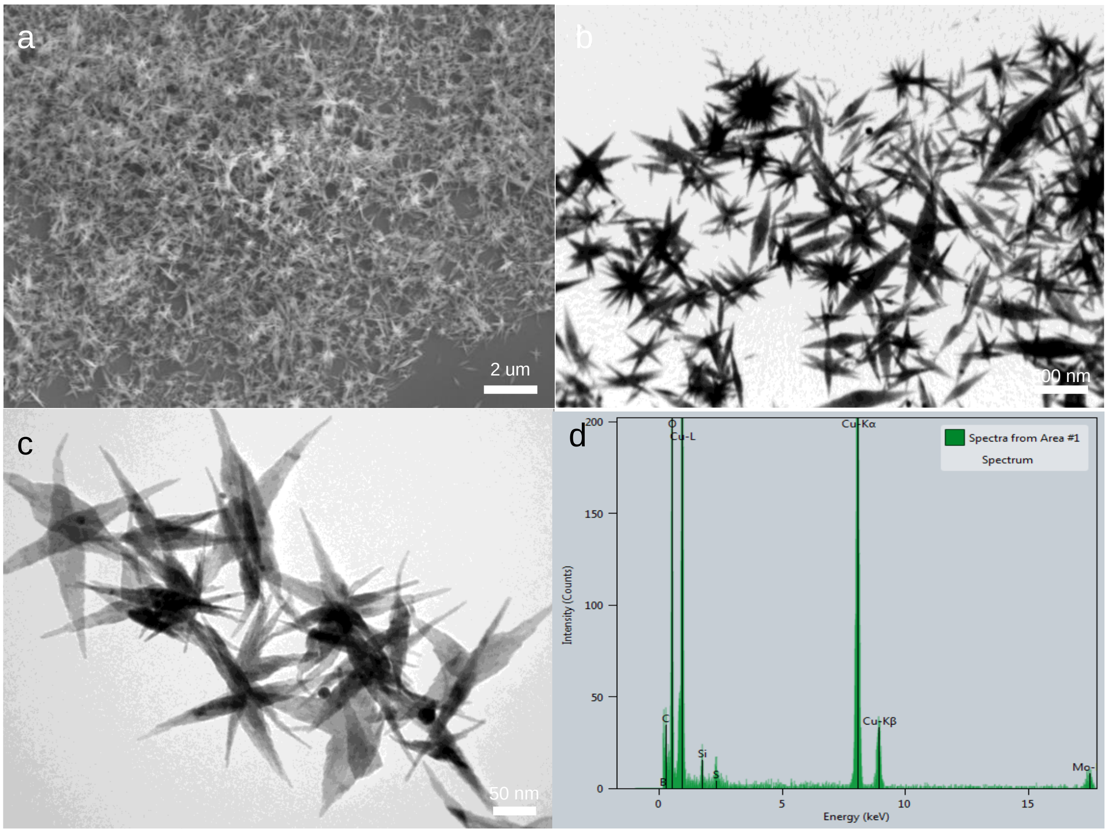

Supplementary Fig. 4. Structural characterization of L-Cu$_x$O-HC. (a) SEM image. (b) Low - magnification and (c) high-magnification TEM images. (d) Typical EDS spectra of L-Cu$_x$O-HC.

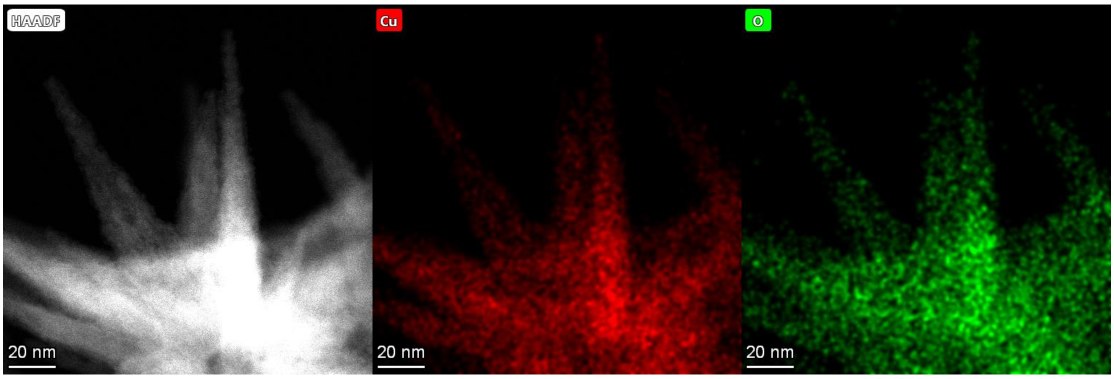

Supplementary Fig. 5. Structural characterization of L-Cu_xO-HC. Energy-dispersive X-ray spectroscopy (EDX) elemental mapping of L-Cu_xO-HC. The results reveal that Cu and O are uniformly distributed in the catalyst.

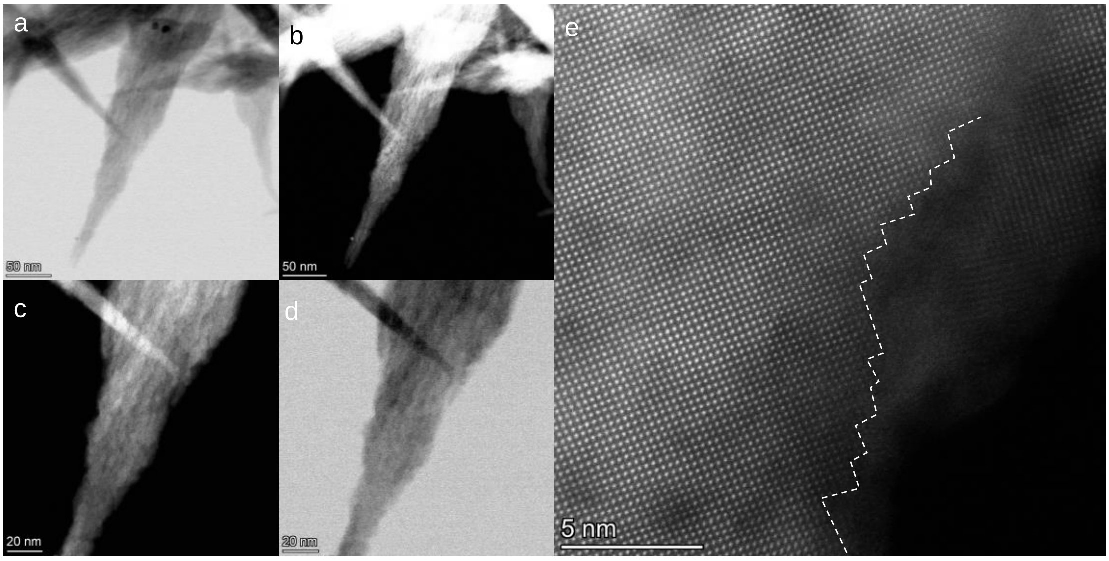

Supplementary Fig. 6. Structural characterization of L-Cu_xO-HC. (a-d) Spherical aberration -corrected high-resolution HAADF-STEM images and (e) STEM of L-Cu_xO-HC. (Dashed polyline is step fault.)

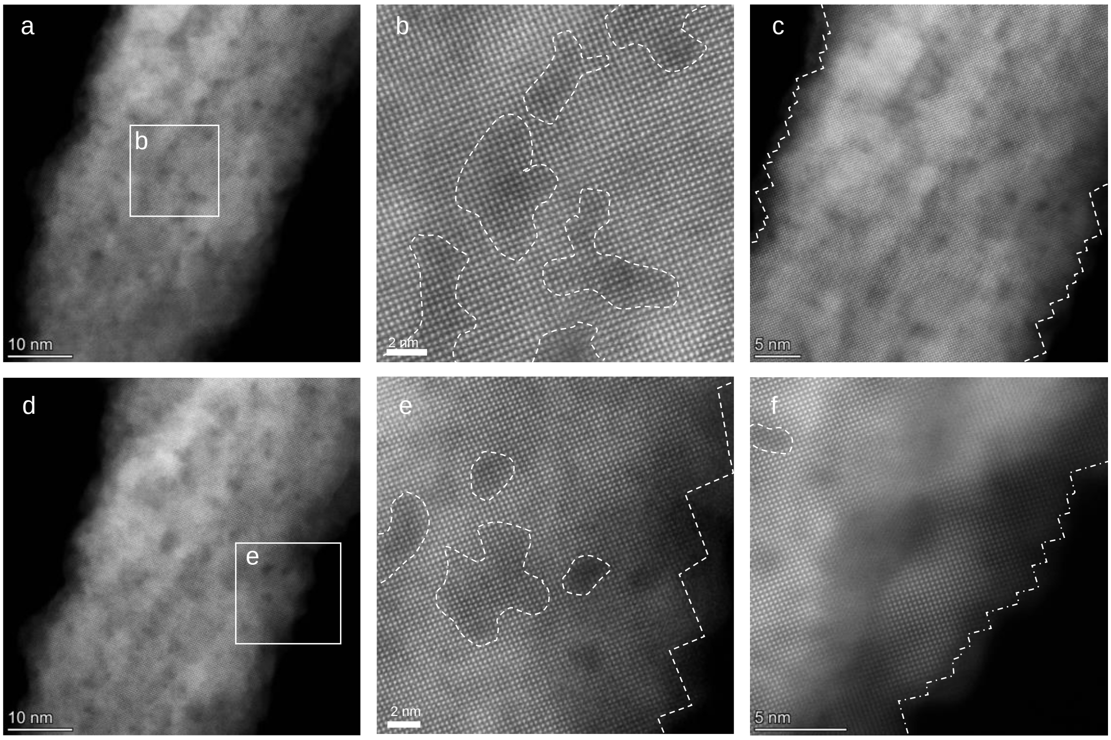

Supplementary Fig. 7. Structural characterization of L-Cu_xO-HC. (a-f) Spherical aberration -corrected high-resolution HAADF-STEM images of L-Cu_xO-HC. (White circles is interface and dashed polyline is step fault.)

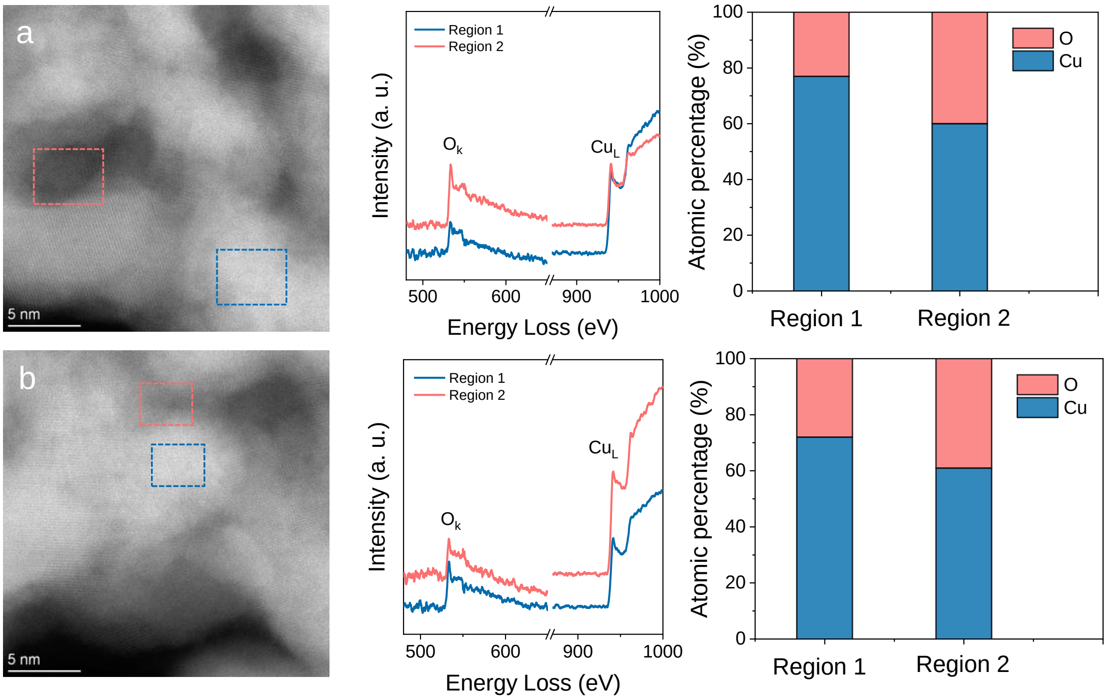

Supplementary Fig. 8. Structural characterization of L-Cu_xO-HC. (a,b) Spherical aberration-corrected high-resolution HAADF-STEM images and the corresponding electron energy loss spectroscopy (EELS) spectra of O K-edge and Cu L-edge and fitted atomic percent of L-Cu_xO-HC.

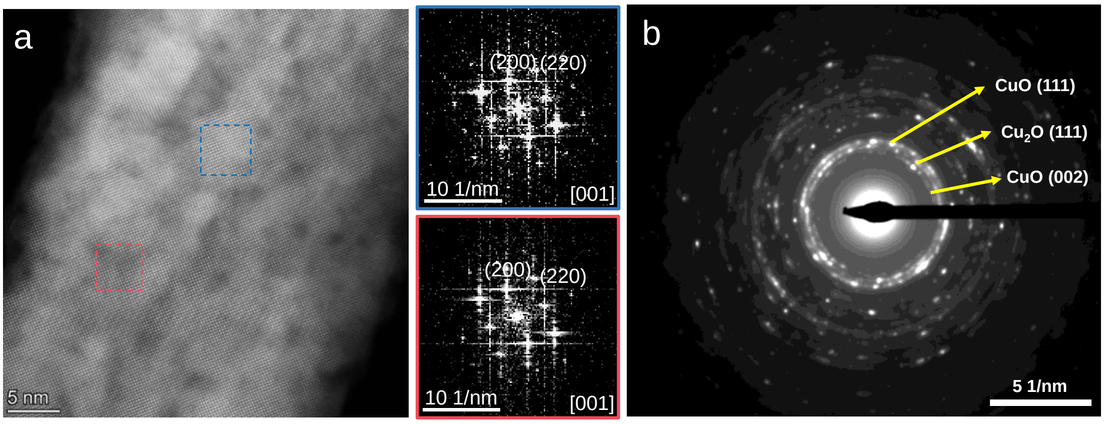

Supplementary Fig. 9. Structural characterization of L-Cu_xO-HC. (a) Spherical aberration -corrected high-resolution HAADF-STEM images and corresponding FFT pattern and (b) selected area electron diffraction (SAED) of L-Cu_xO-HC.

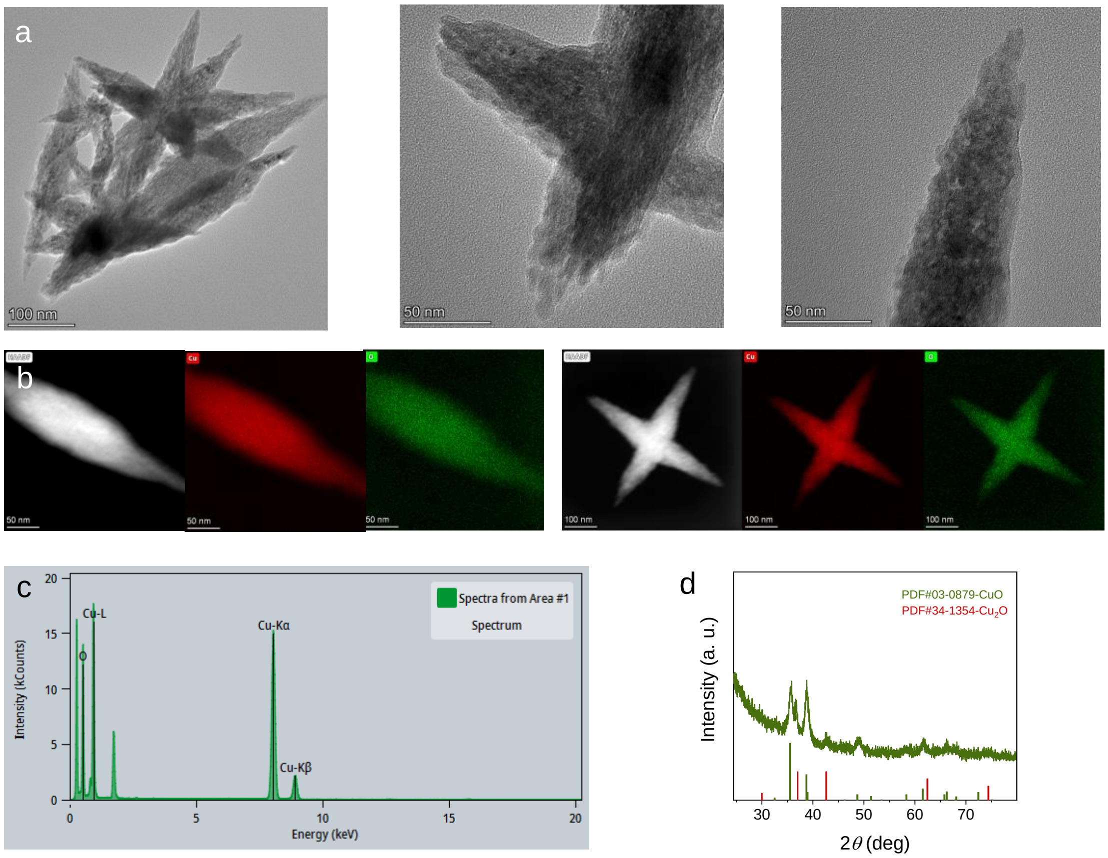

Supplementary Fig. 10. Structural characterization of L-Cu_xO-MC. (a) Low-magnification TEM images. (b) EDX elemental mapping images. (c) Typical EDS spectra and (d) XRD pattern of L-Cu_xO-MC.

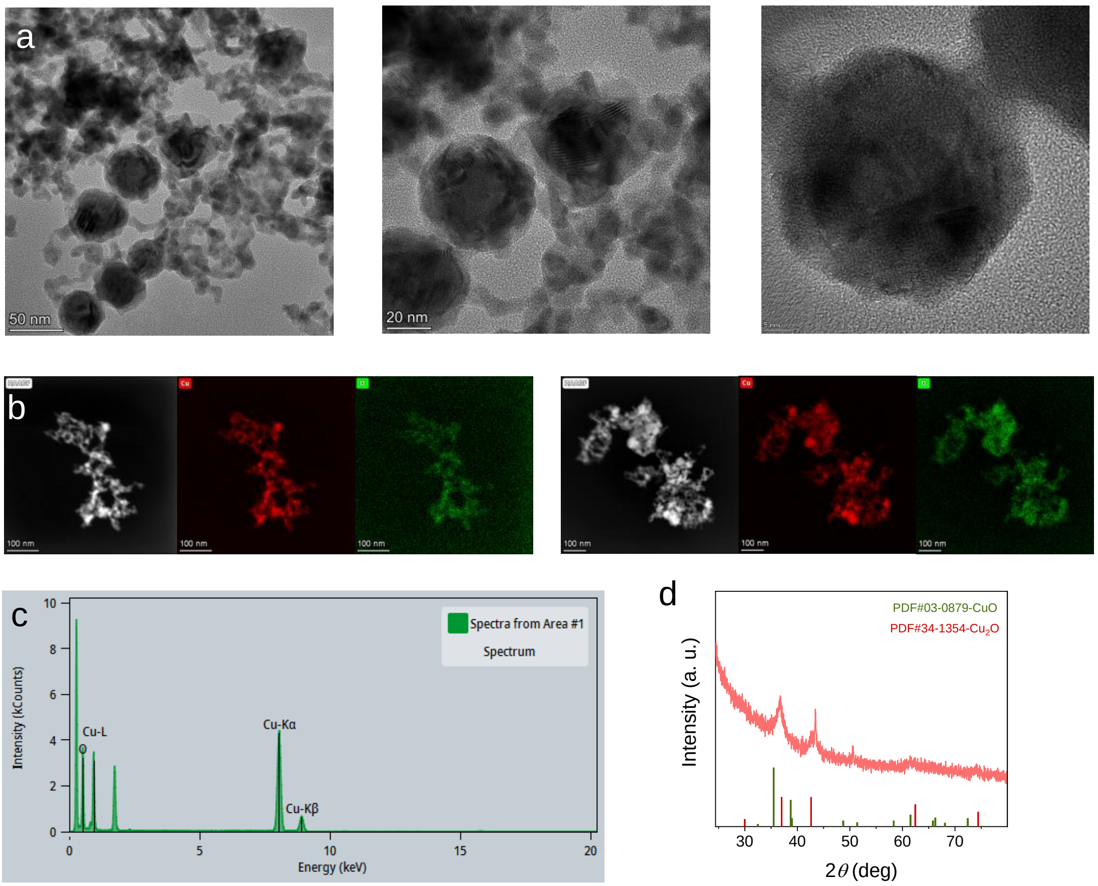

Supplementary Fig. 11. Structural characterization of L-Cu_xO-LC. (a) Low-magnification TEM images. (b) EDX elemental mapping images. (c) Typical EDS spectra and (d) XRD pattern of L-Cu_xO-LC.

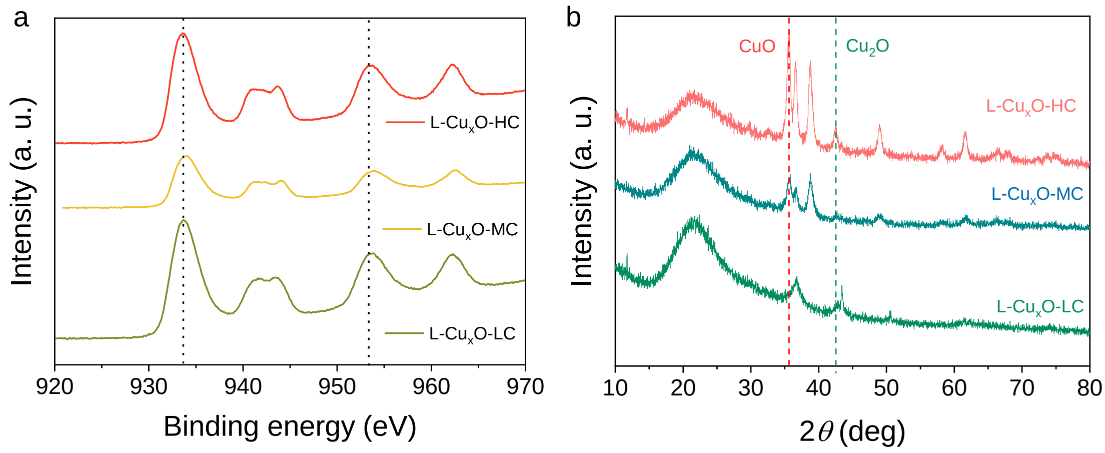

Supplementary Fig. 12. Structural characterization of L-Cu_xO with different tip angles. (a) XPS spectra and (b) XRD pattern of L-Cu_xO-HC, L-Cu_xO-MC and L-Cu_xO-LC.

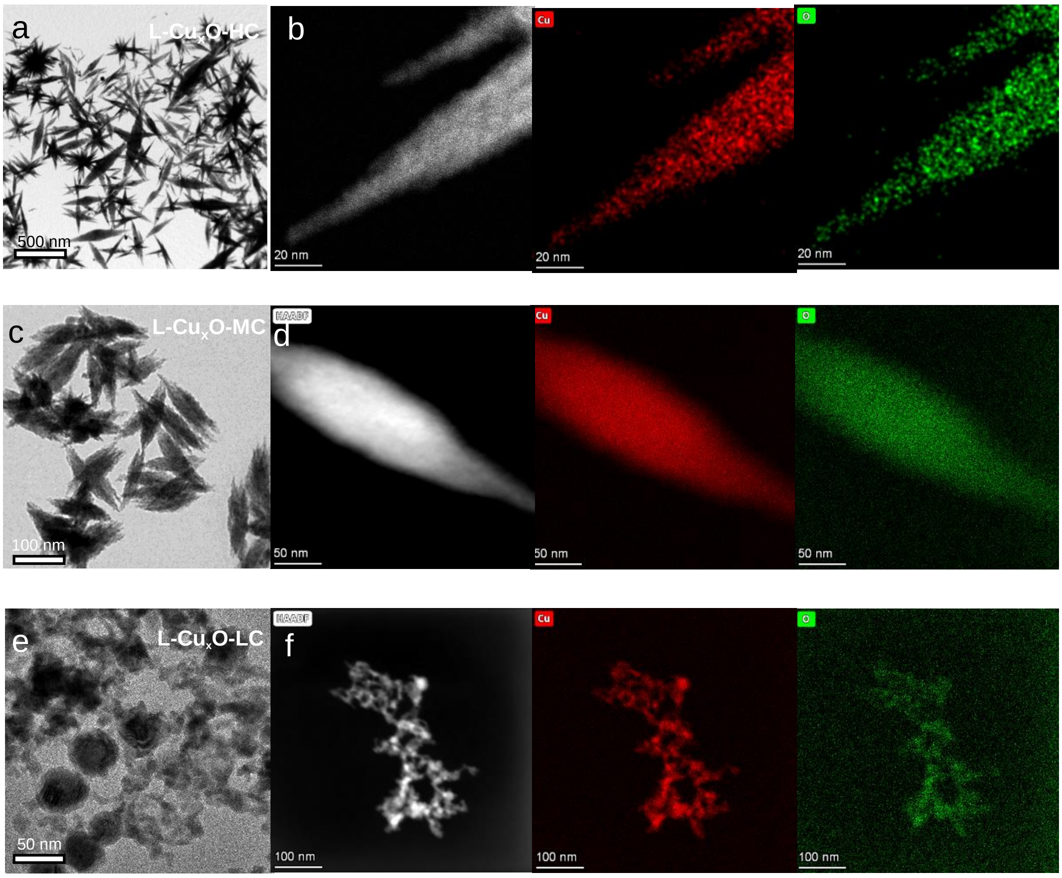

Supplementary Fig. 13. Structural characterization of L-Cu_xO with different tip angles. (a, c, e) TEM images and (b, d, f) EDX mapping images of L-Cu_xO-HC, L-Cu_xO-MC and L-Cu_xO-LC, respectively.

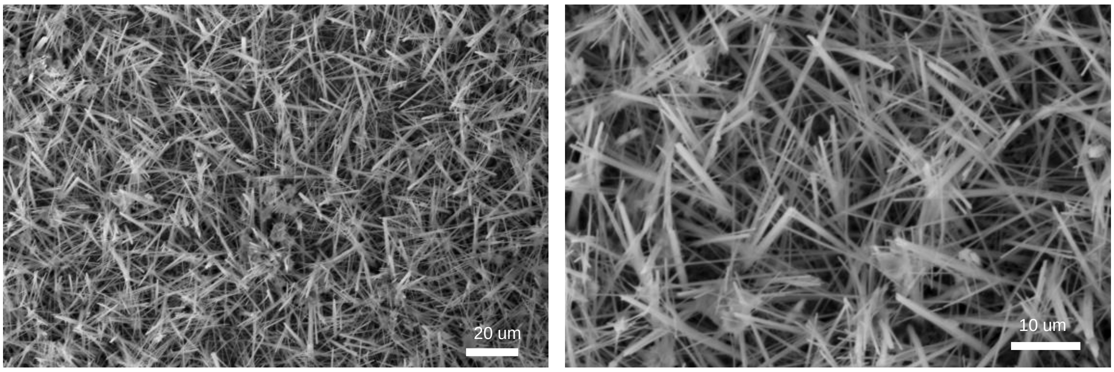

Supplementary Fig. 14. Structural characterization of Cu-TIP. SEM images of Cu-TIP.

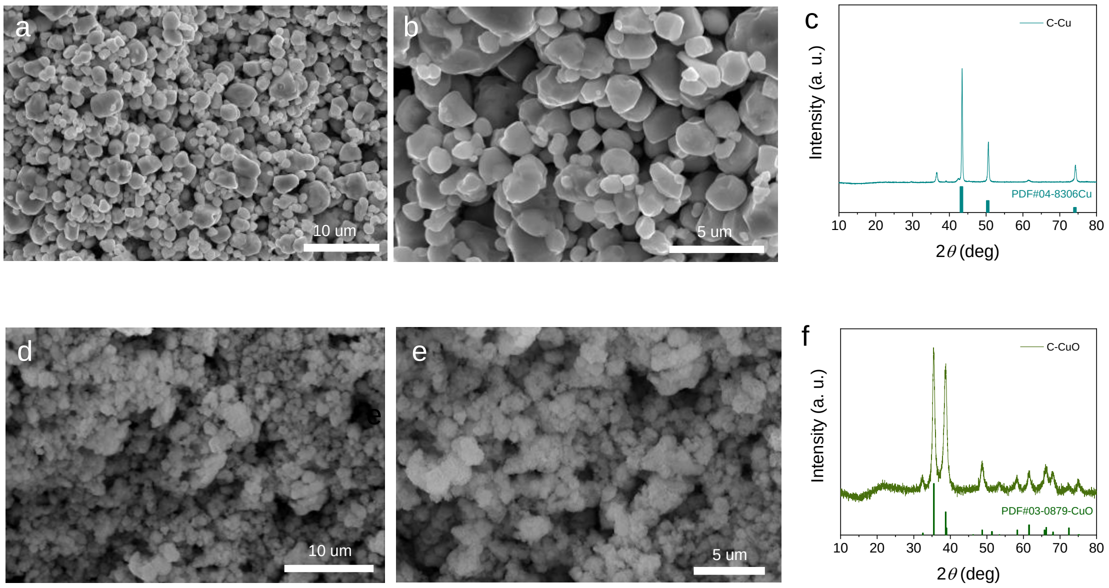

Supplementary Fig. 15. Structural characterization of C-Cu and C-CuO. (a, b) SEM images and (c) XRD pattern of C-Cu. (d, e) SEM images and (f) XRD pattern of C-CuO.

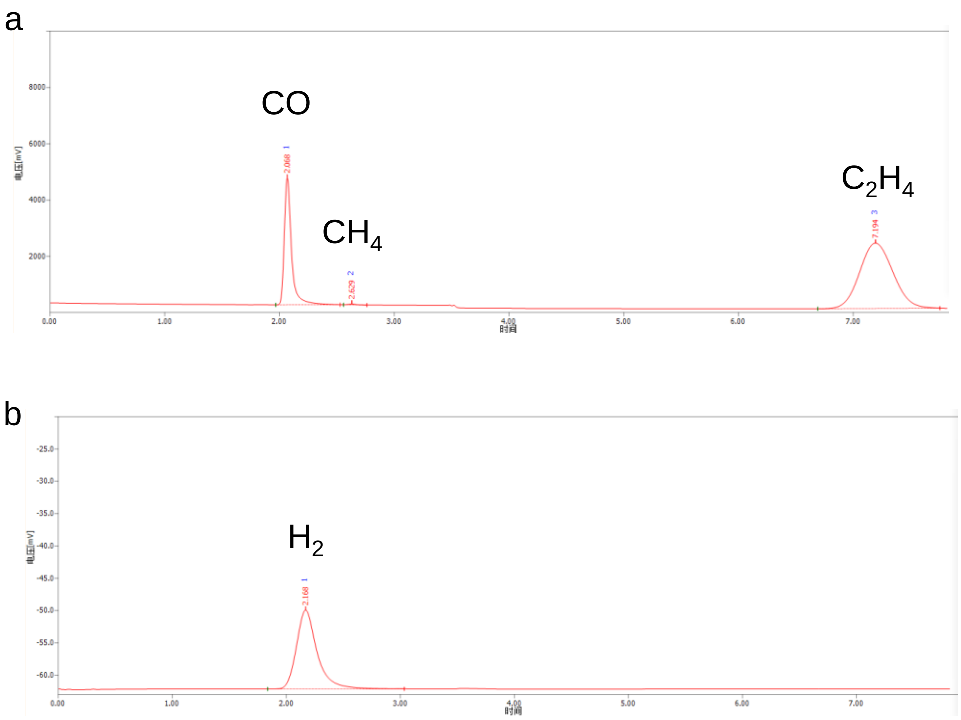

Supplementary Fig. 16. Representative GC traces of the gas products (CO, C_2H_4, CH_4 and H_2). (a) FID detector traces and (b) TCD detector traces form GC.

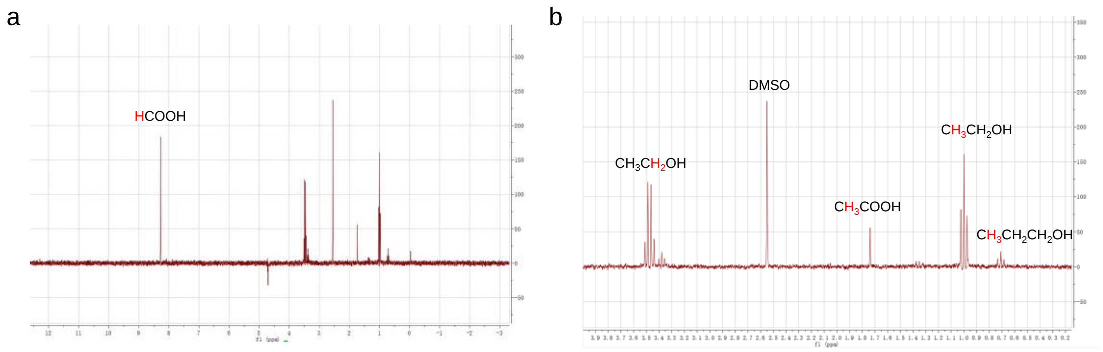

Supplementary Fig. 17. Representative NMR spectra of liquid products. (a) Representative ^{1}H-NMR spectrum of liquid products. (b) Representative ^{1}H-NMR spectrum of liquid products enlarges form (a).

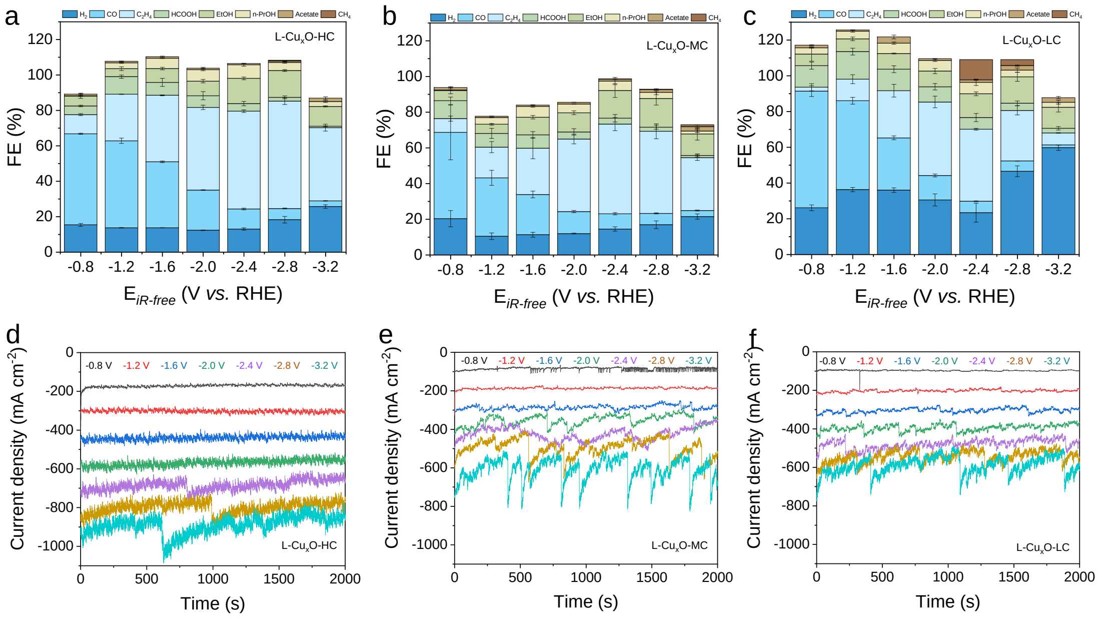

Supplementary Fig. 18. CO_2RR performance in flow cell. (a-c) FE of different products during CO_2RR and (d-f) Chronoamperometry curves at different potentials of L-Cu_xO-HC, L-Cu_xO-MC and L-Cu_xO-LC, respectively. The error bars indicate standard deviation among values from three repeated measurements.

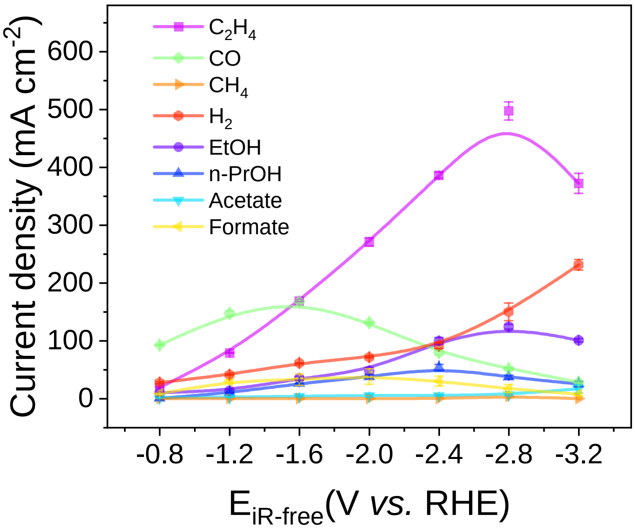

Supplementary Fig. 19. CO_2RR performance in flow cell. Current density of each CO_2RR product and H_2 on L-Cu_xO-HC at different applied potentials in 1 M KOH (without iR-correction). The error bars indicate standard deviation among values from three repeated measurements.

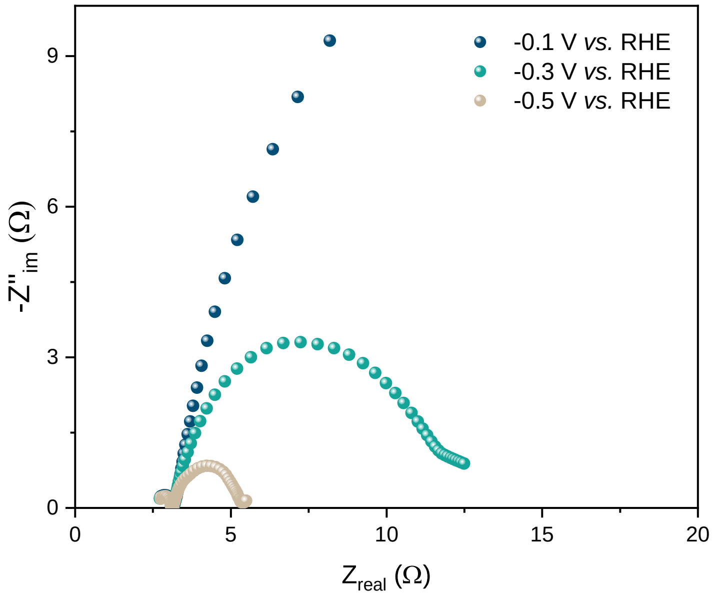

Supplementary Fig. 20. Electrochemical characterization. EIS measurement of L-Cu_xO-HC at different potentials in 1 M KOH electrolyte with a scanning frequency range from 1000 kHz to 0.1 Hz.

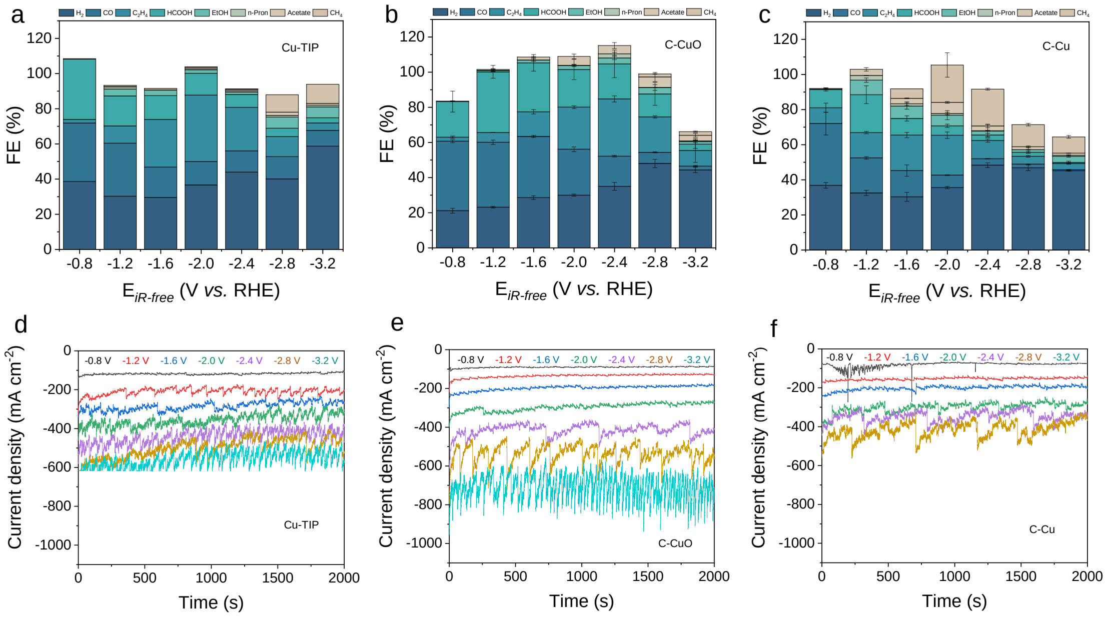

Supplementary Fig. 21. CO2RR performance in flow cell. (a-c) FE of different products during CO2RR and (d-f) Chronoamperometry curves at different potentials of Cu-TIP, C-CuO and C-Cu, respectively. The error bars indicate standard deviation among values from three repeated measurements.

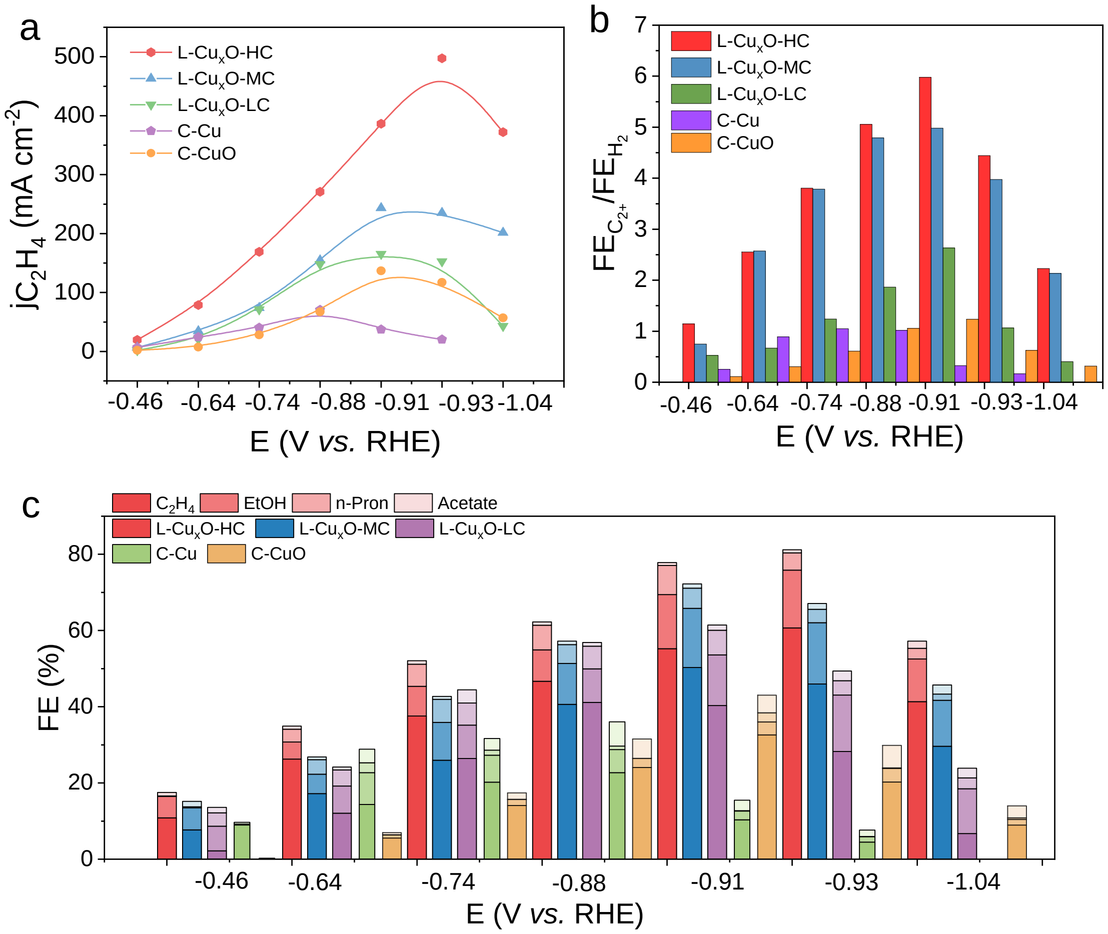

Supplementary Fig. 22. CO_2RR performance in flow cell at different potential with 80% iR-compensation. (a) C_2H_4 Current density of different catalysts. (b) Ratio of FE_{C2+}/FE_{H2} at different potential and (c) FE of different C_{2+} products during CO_2RR at different potentials, respectively.

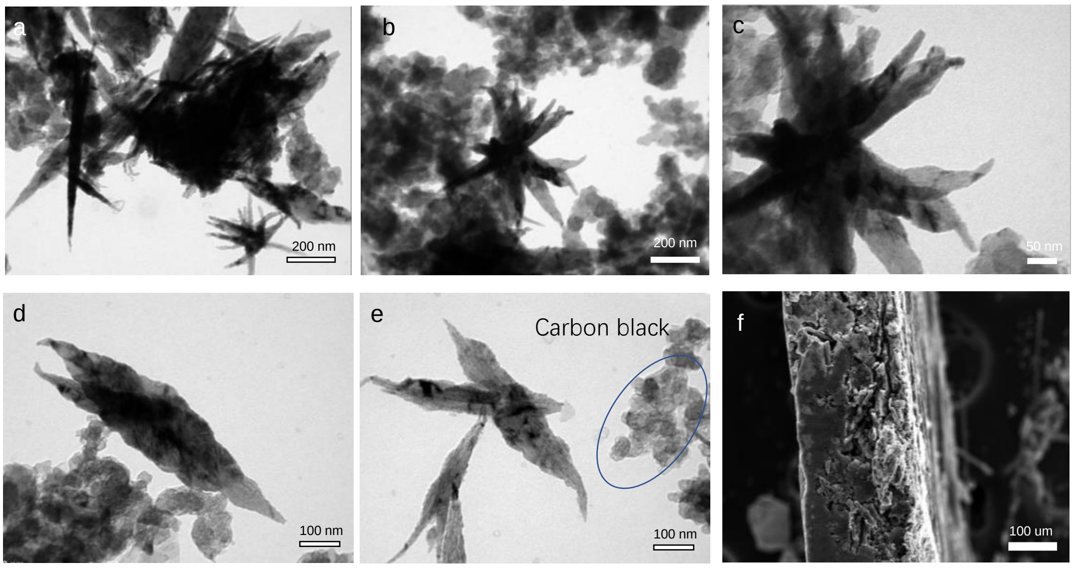

Supplementary Fig. 23. Characterizations of the tested L-CuxO-HC. (a-e) TEM images and (f) SEM image of L-CuxO-HC after stability test. (Blue circle is the carbon black from GDL)

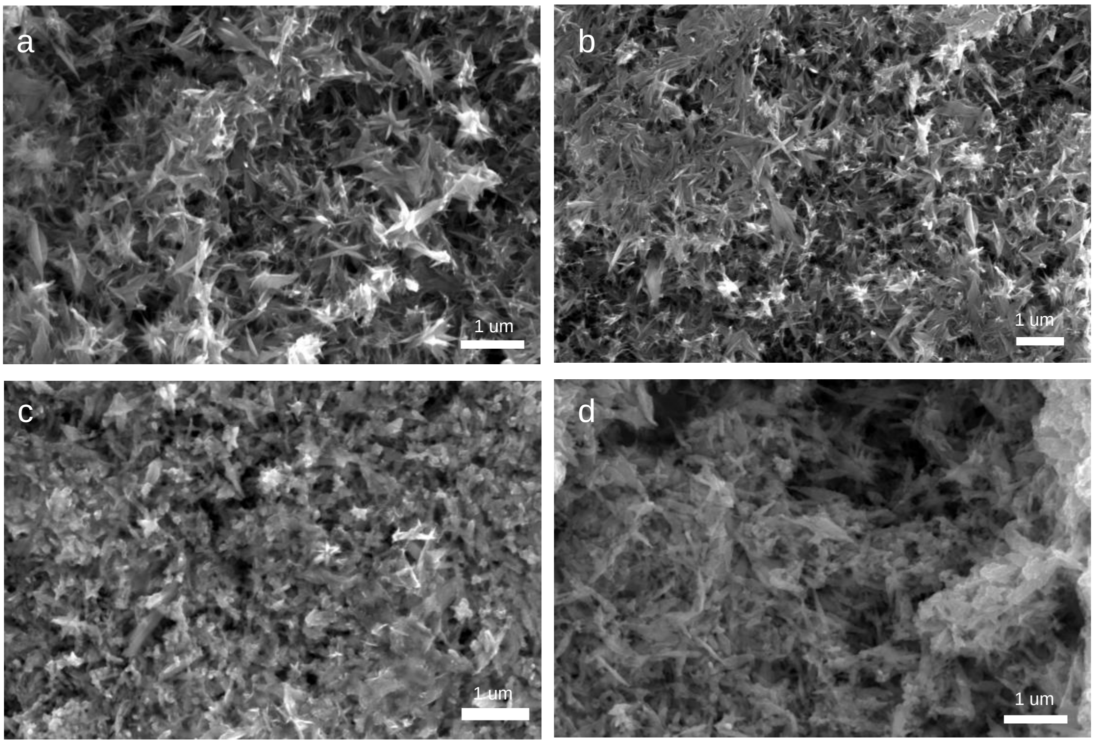

Supplementary Fig. 24. Characterizations of the tested L-CuxO-HC. (a-d) SEM images of L-CuxO-HC after stability test.

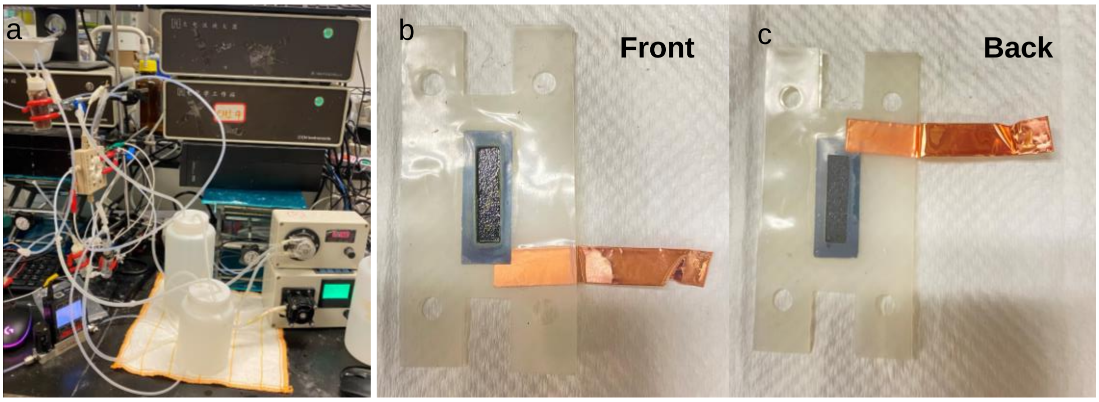

Supplementary Fig. 25. Optical photograph of the electrochemical process over CO_2RR. (a) Photography of the flow cell device. (b) Front and (c) back of GDL loading with L-Cu_xO-HC after electrochemical CO_2.

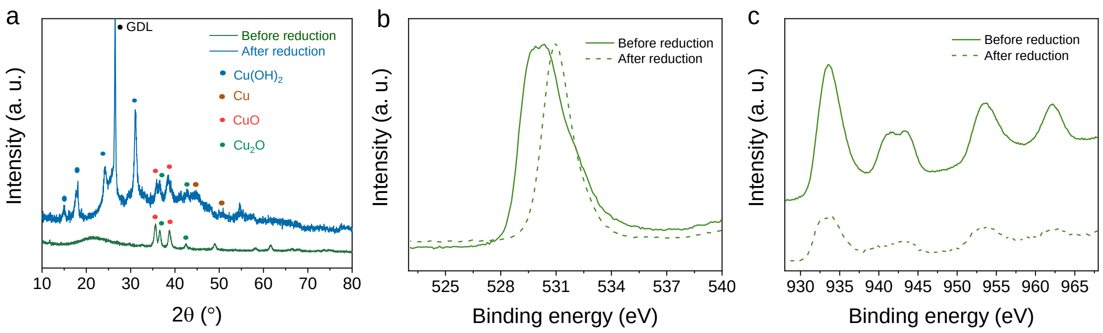

Supplementary Fig. 26. Characterizations of the tested L-Cu_xO-HC. (a) XRD pattern. (b) O 1*s* XPS spectra and (c) Cu 2*p* XPS spectra of L-Cu_xO-HC before and after stability test.

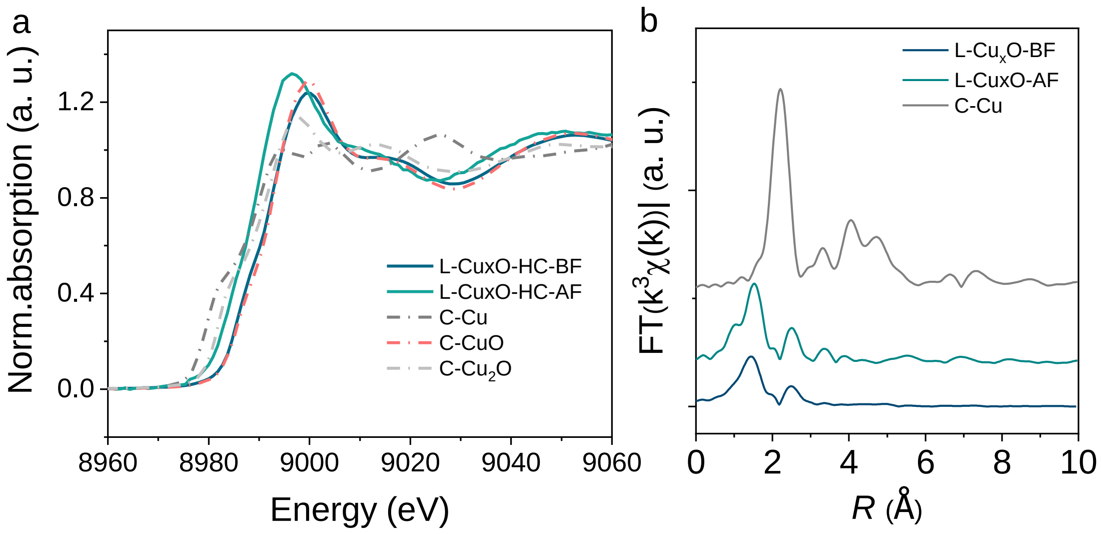

Supplementary Fig. 27. Characterizations of the tested L-Cu_xO-HC. (a) Normalized XANES, (b) Fourier transformed EXAFS of L-Cu_xO-HC before and after CO_2RR.

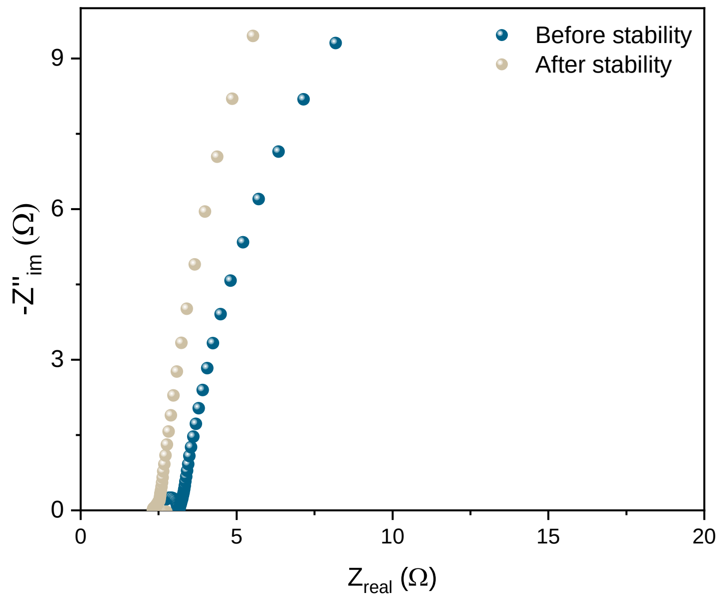

Supplementary Fig. 28. Electrochemical characterization. EIS measurement before and after stability in L-Cu_xO-HC taken at -0.1 V vs. RHE in 1 M KOH electrolyte with a scanning frequency range from 1000 kHz to 0.1 Hz.

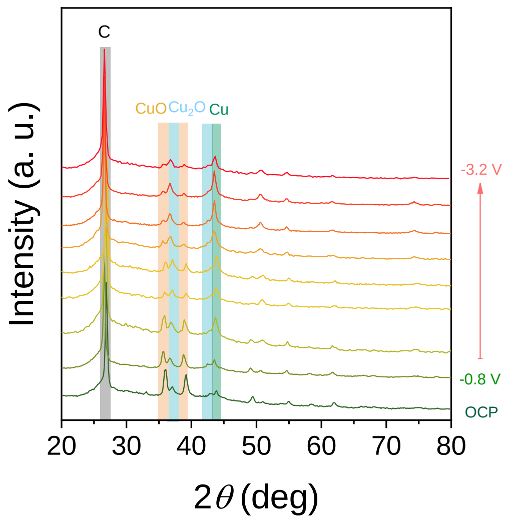

Supplementary Fig. 29. X-ray diffraction (XRD) study in 1 M KOH electrolyte with flow cell. A customized electrochemical cell was used with a Pt wire and an Ag/AgCl electrode as the counter and reference electrodes, respectively. The cathode and anode compartments were separated using an AEM membrane. The catalysts were dropped onto gas diffusion electrode as the working electrode. The measurements were conducted in open circuit potential and from −0.8 V to -3.2 V vs. RHE without iR correction. The XRD of the working electrodes was recorded immediately after the electrolysis at each potential

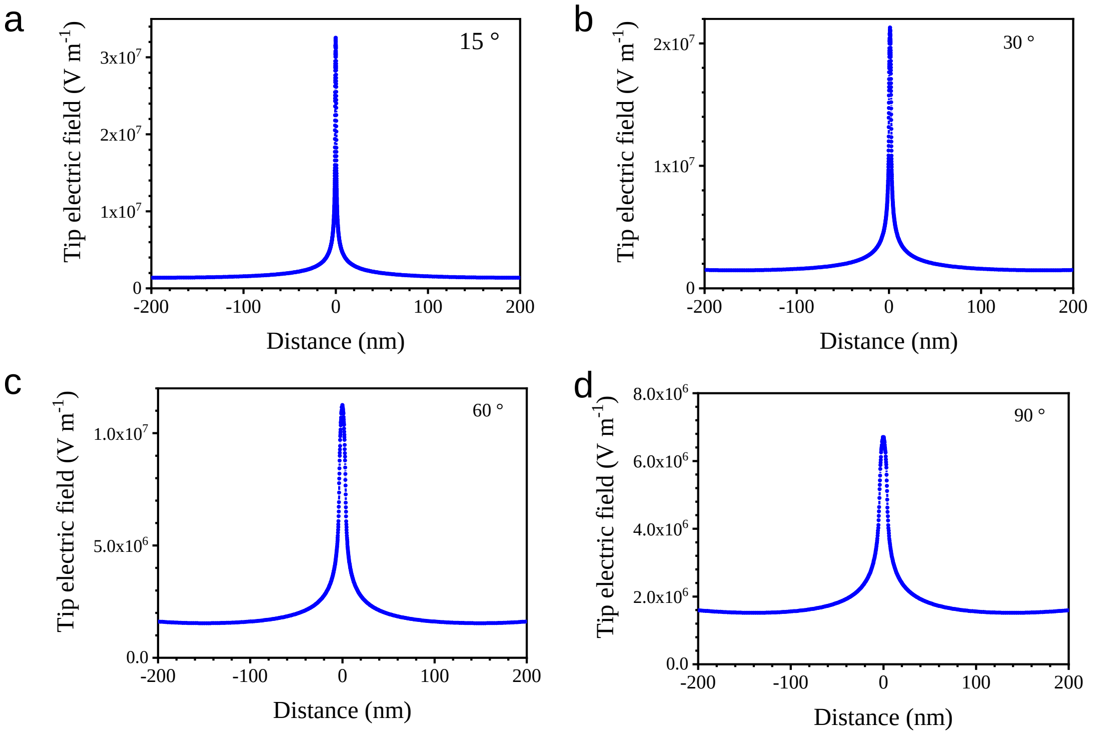

Supplementary Fig. 30. Theoretical Simulations. Electric field intensity distribution on the electrode surface when the tip angle is (a) 15°, (b) 30°, (c) 60°, (d) 90°.

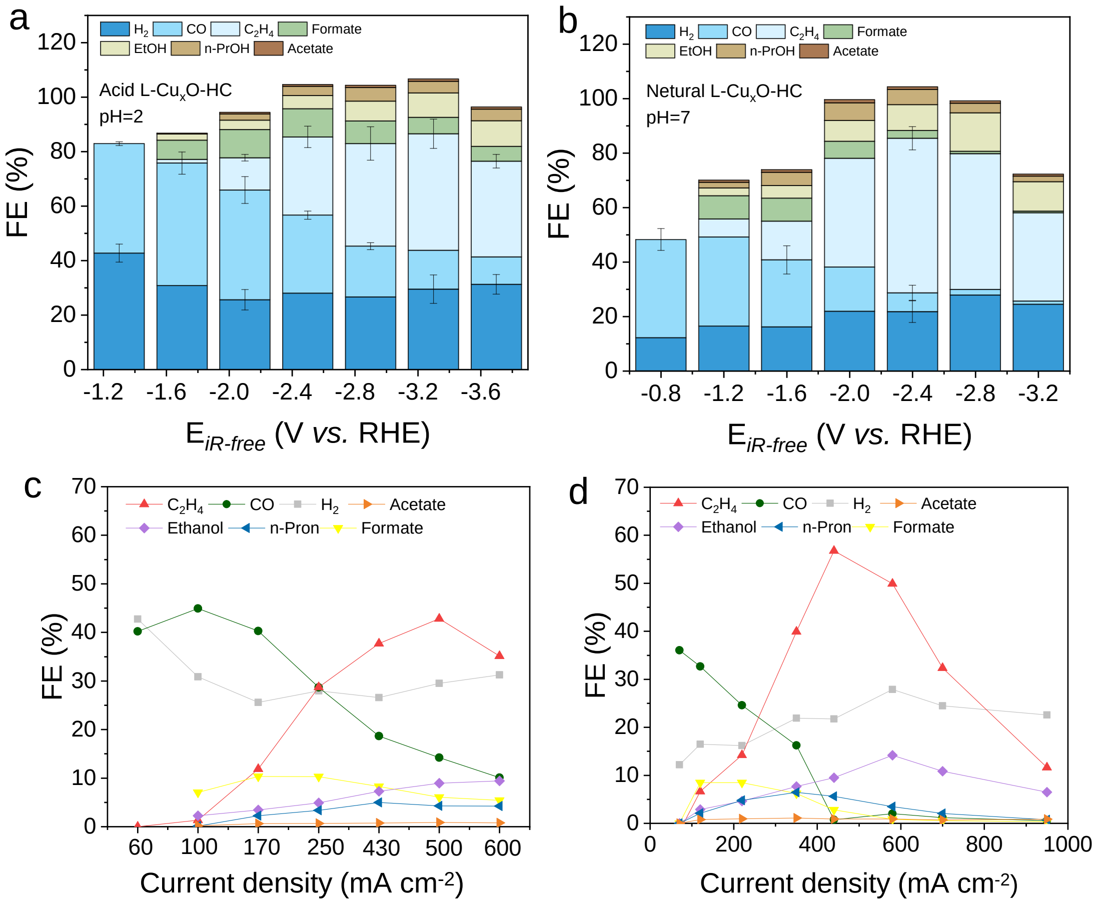

Supplementary Fig. 31. CO_2RR performance in flow cell. (a, b) FE of different products during CO_2RR at different potentials and (c, d) FE of different products at different current density of L-Cu_xO-HC in acid and neutral, respectively. The error bars indicate standard deviation among values from three repeated measurements.

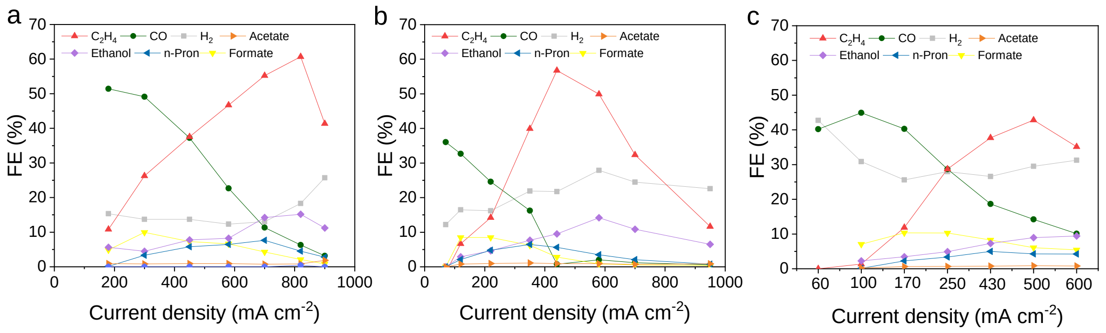

Supplementary Fig. 32. CO_2RR performance in flow cell at different current density. (a-c) FE of different products during CO_2RR at different current density in (a) alkaline, (b) neutral and (c) acid environment, respectively.

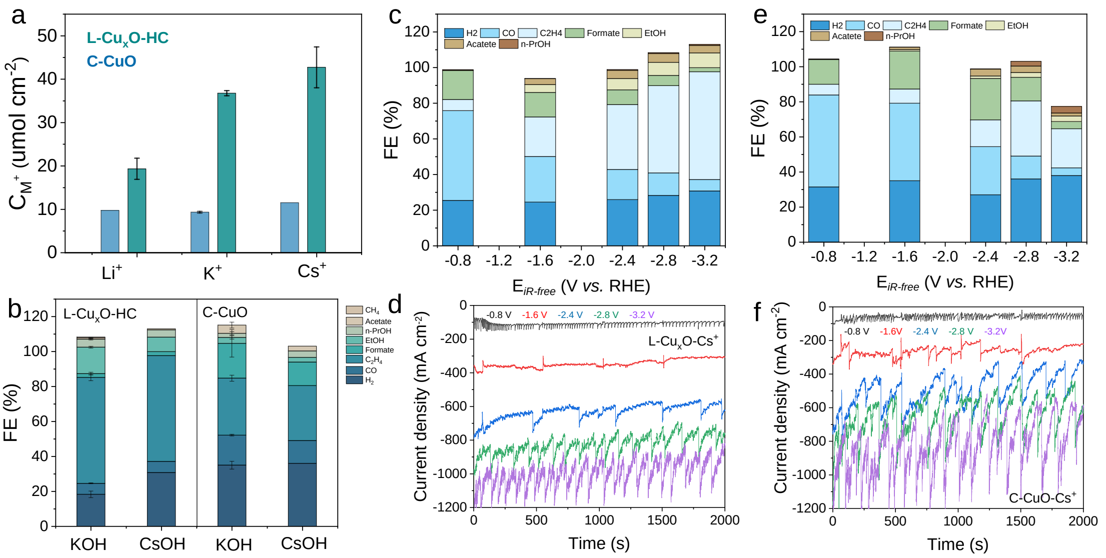

Supplementary Fig. 33. Research on the effect of electric field on cations. (a) Cation adsorption experiment and (b) FE of each product in electrolytes with different cations of L-Cu_xO-HC and C-CuO at their best potential; (c, e) FE of each product at different potentials and (d, f) the corresponding i-t curves of L-Cu_xO-HC and C-CuO in electrolytes with Cs^+ cations, respectively. The error bars indicate standard deviation among values from three repeated measurements.

We conducted the adsorption reactions of lithium ions and cesium ions separately on L-Cu_xO-HC (high electric field) and C-CuO (low electric field). According to the results of cation adsorption experiments, the strong electrostatic field at the tip curvature could increase the accumulation of different cations with 2-4 times, and meanwhile, the electrostatic field at the tip correlates with cations (Supplementary Fig. 33). Then we conducted their carbon dioxide catalytic performance in different solutions. However, in lithium hydroxide solution, due to severe flooding and hydrogen production, gas products could not be collected in the online testing system and there was almost no liquid product according to NMR result, so we only compared the catalytic performance with potassium and cesium ions. It is found that in potassium hydroxide or cesium hydroxide solutions, the FE of the product does not change much for L-Cu_xO-HC and C-CuO. In terms of current density, the current density of C-CuO in cesium hydroxide increases compared to that in potassium hydroxide, possibly due to the cationic effect. This is mainly because the corrected Stokes radius of hydrated cations show a decreasing trend from Li^+ to K^+ and Cs^+, which is contrary to that of the ionic radius of cations (Supplementary Table 1). The difference in Stokes radius can lead to a varied change in the total amount of cations in the vicinity of the electrode and the electric field at the tip curvature will intensify the difference in the total amount of cation adsorption, resulting in different CO_2RR performances[1-4].

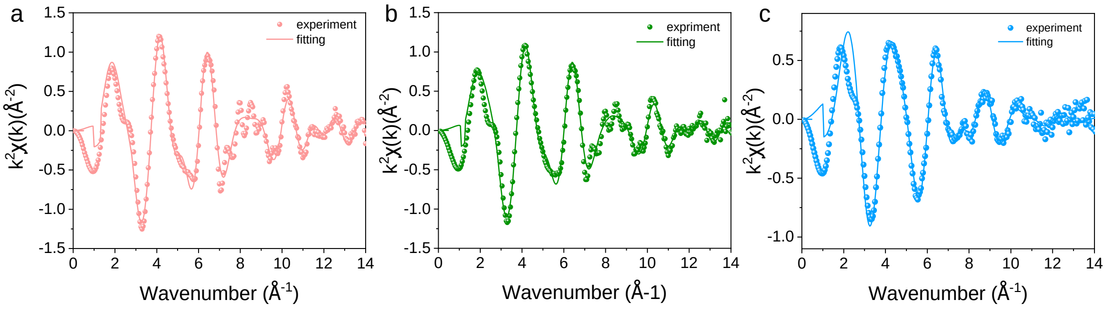

Supplementary Fig. 34. Fitting results of Cu K-edge EXAFS spectra of different catalysts. Fourier transform of Cu-K edge EXAFS fitting results of (a) L-Cu_xO-HC, (b) L-Cu_xO-MC and (c) L-Cu_xO-LC, respectively.

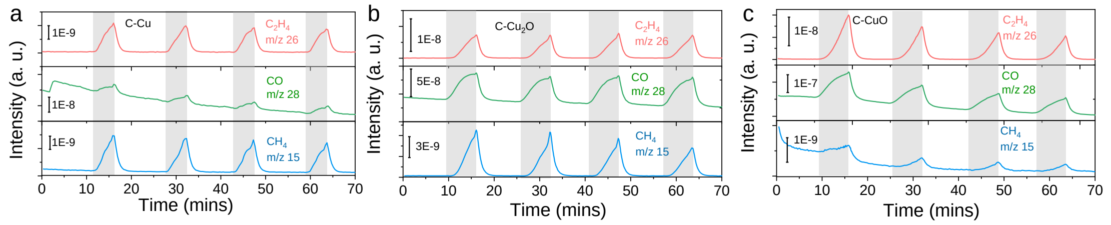

Supplementary Fig. 35. Product analysis. Online DEMS over (a) C-Cu (b) C-Cu_2O and (c) C-CuO, respectively. Reliable onset potential evolutions of a variety of CO_2RR products on commercial Cu, Cu_2O and CuO have not been reported before. We noted that CO, C_2H_4 and CH_4 generated in a different order with different Cu oxygen content during CO_2RR process. From result of DEMS on commercial Cu, Cu_2O and CuO, we find methane is gradually difficult to produce with oxygen content increase from Cu to CuO, while C_2H_4 are easily to produce.

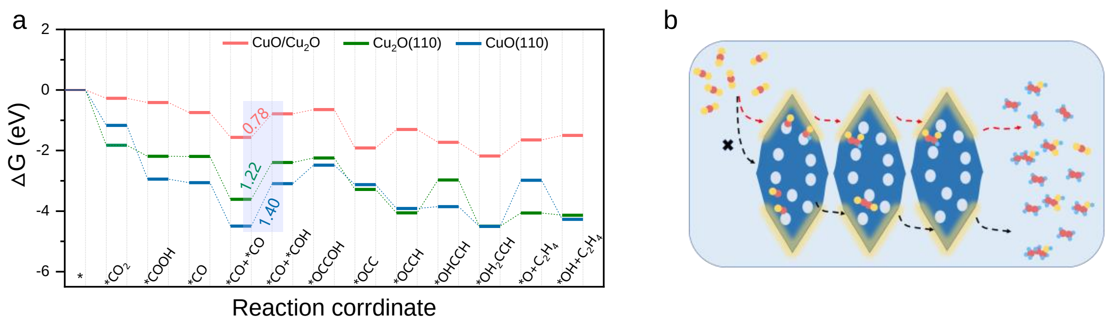

Supplementary Fig. 36. DFT simulation. (a) A reaction energy diagram for the CO_2RR to C_2H_4 on the $Cu_2O(110)$ slab, Cu_2O/CuO interface and $CuO(110)$ slab. (b) schematic illustration. Red: carbon; yellow: oxgyen; blue: hydrogen.

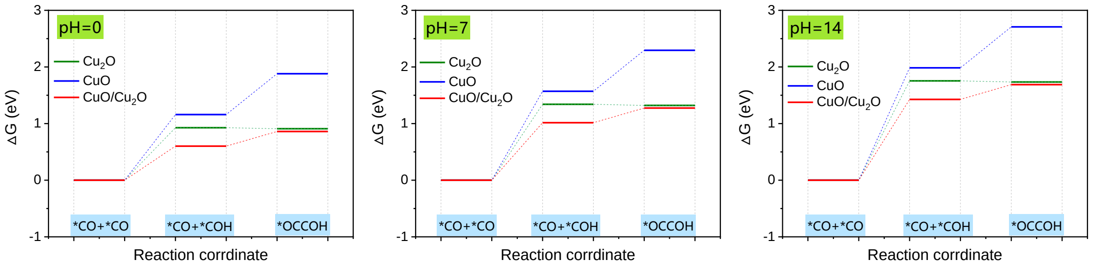

Supplementary Fig. 37. DFT simulation. Reaction Gibbs free energy profile for *CO+*CO→*CO+ *COH→*OCCOH steps on Cu_2O, CuO, and the Cu_2O/CuO model at various pH.

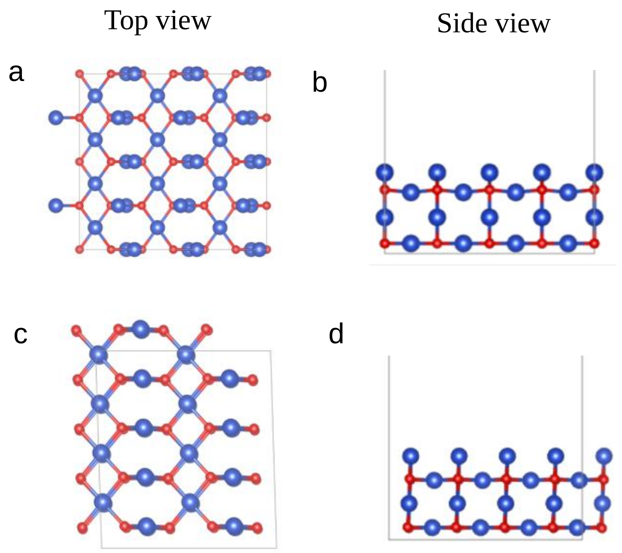

Supplementary Fig. 38. DFT simulation. Optimized geometry of $Cu_2O(110)$ at (a) top view and (b) side view and of CuO(110) slab at (c) top view and (d) side view.

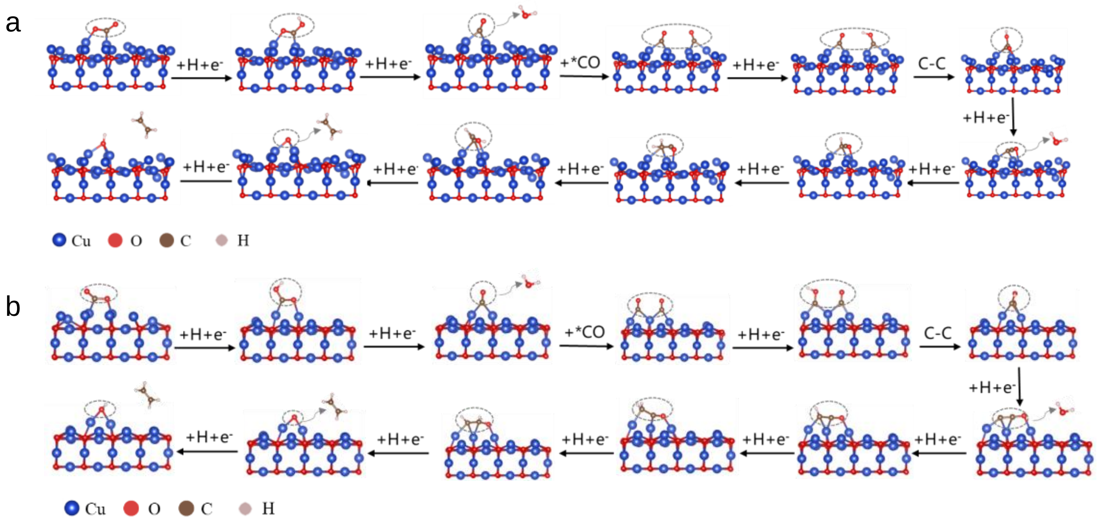

Supplementary Fig. 39. DFT simulation. Optimized structures for the reaction intermediates of the C_2H_4 formation pathway on the (a) $Cu_2O(110)$ slab and (b) CuO(110) slab.

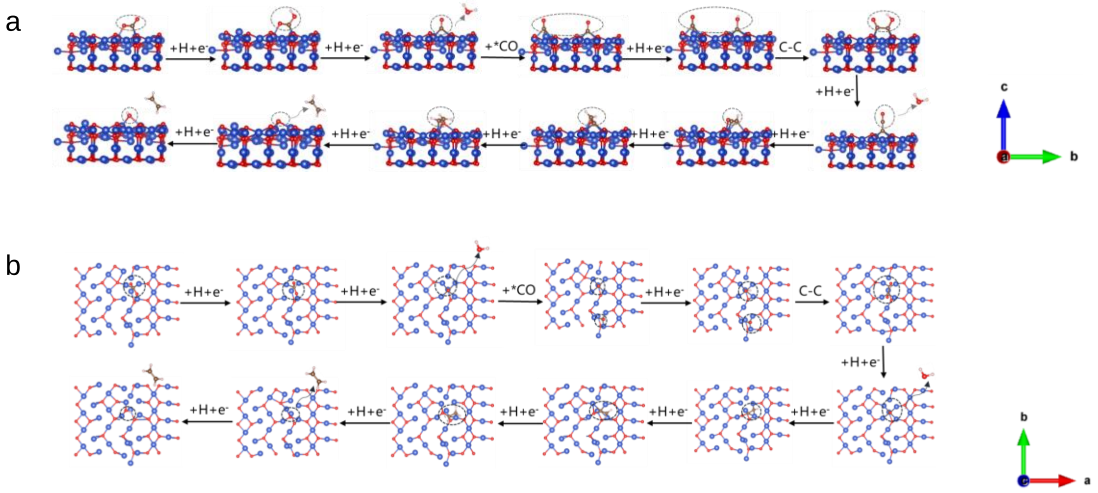

Supplementary Fig. 40. DFT simulation. Optimized structures for the reaction intermediates of the C_2H_4 formation pathway on the CuO/Cu_2O interface: (a) at side view and (b) at top view (only the top layer atoms are shown).

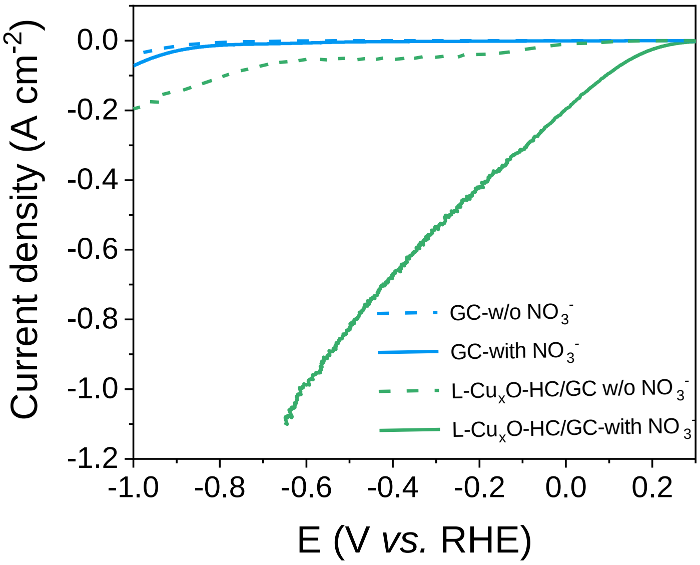

Supplementary Fig. 41. Electrochemical performances. Linear scan voltammetry curves of L-Cu_xO-HC and glass carbon in 1 M KOH and 1 M KOH + 1 M KNO_3 at different potential with 50% iR-compensation.

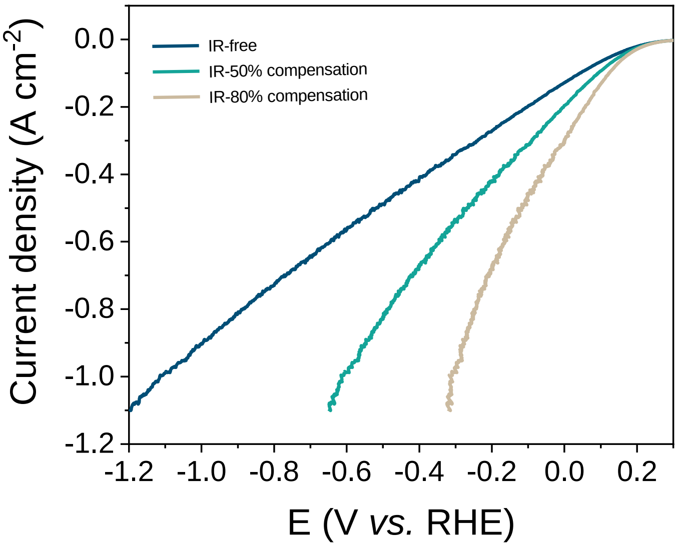

Supplementary Fig. 42. Electrochemical performances. Linear scan voltammetry curves of L-Cu_xO-HC in 1 M KOH + 1 M KNO_3 at different potential with iR-free, iR-50% compensation and iR-80% compensation.

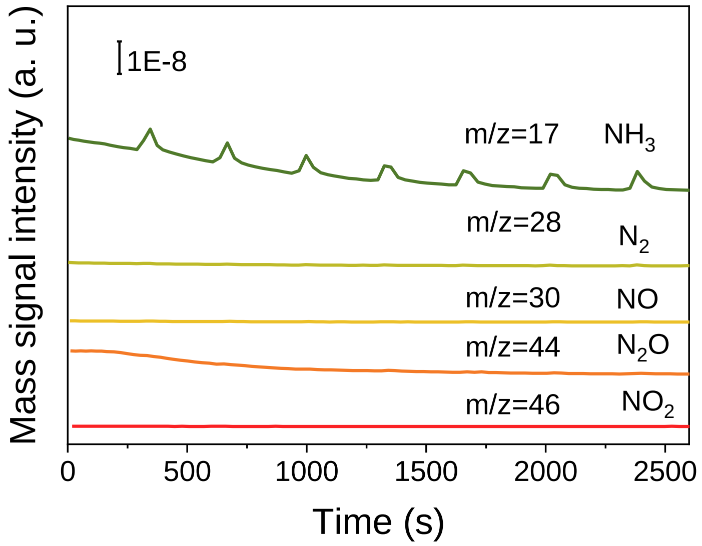

Supplementary Fig. 43. The OEMS result. The OEMS result for the possible gaseous products of NITRR (NH_3 (g), N_2, NO, N_2O, NO_2).

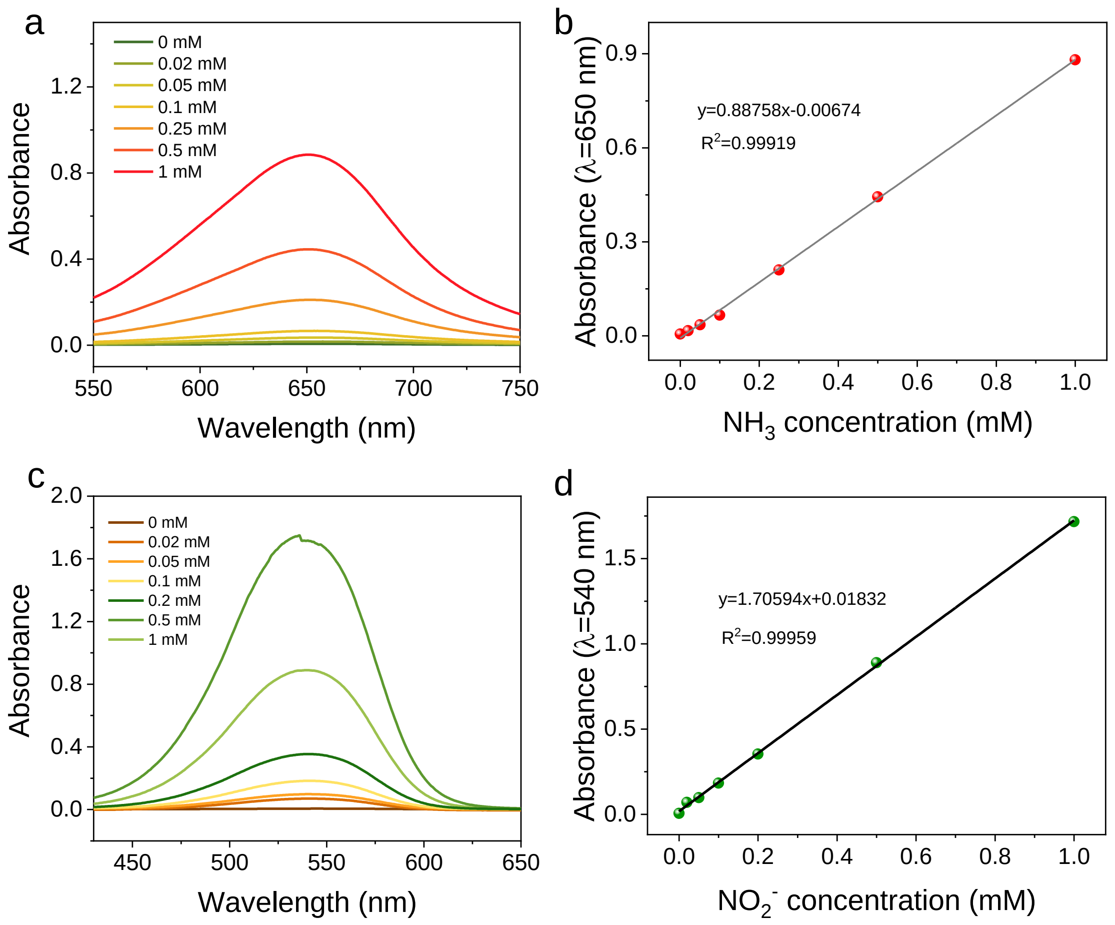

Supplementary Fig. 44. Calibration curves. UV-vis calibration curves of (a, b) ammonia (NH_3) and (c, d) nitrite (NO_2^-), respectively.

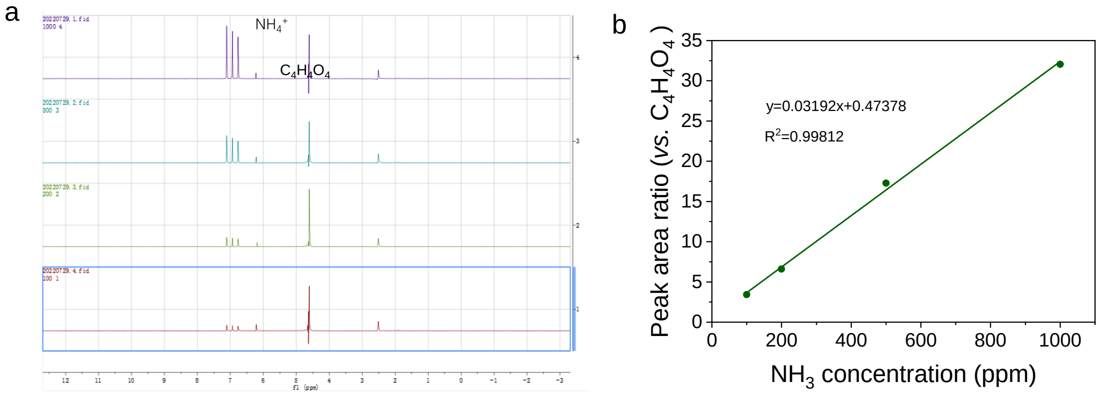

Supplementary Fig. 45. **Calibration curves.** (a) ^{1}H NMR calibration curve of NH_3 using different ammonium chloride concentration solutions of known concentration as standards. (b) ^{1}H NMR calibration curve of NH_3.

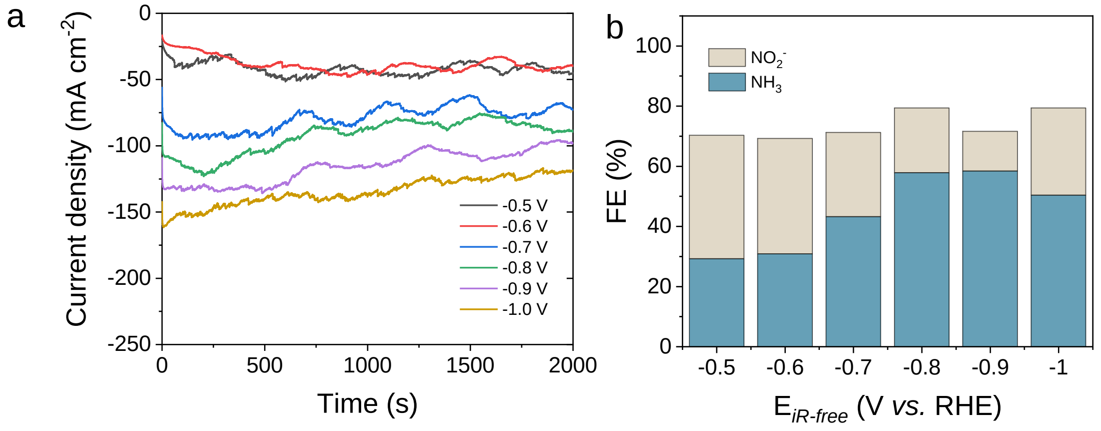

Supplementary Fig. 46. Electrochemical performances. (a) Chronoamperometry and (b) FE of NH_3 and NO_2^- on glass carbon at different potential in 1 M KOH + 1 M KNO_3.

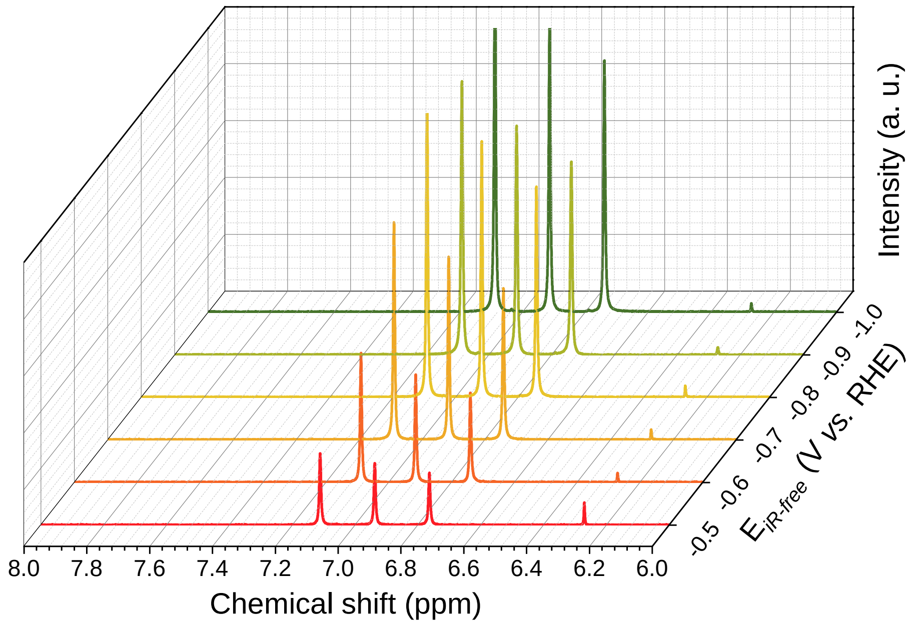

Supplementary Fig. 47. Product analysis. ^{1}H NMR calibration curve of NH_3 at different potential.

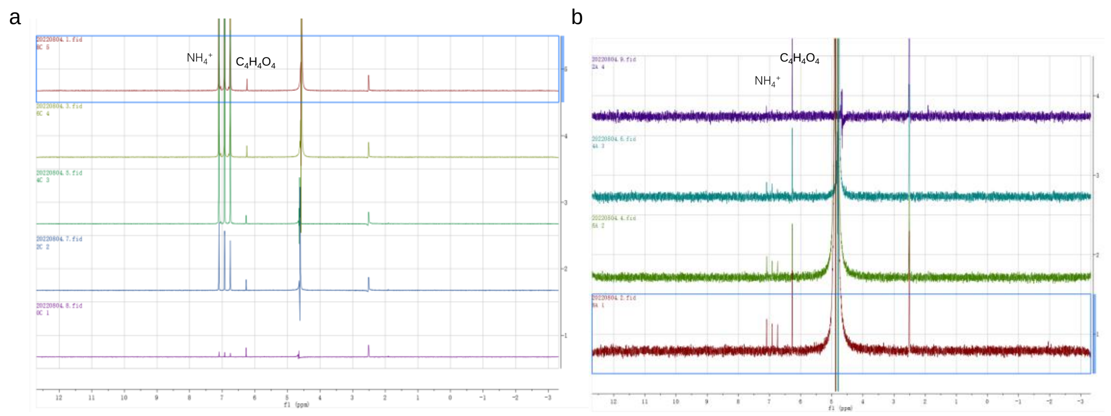

Supplementary Fig. 48. **Product analysis.** (a) Representative ^{1}H-NMR spectrum of liquid products collected from the cathode side. (b) Representative ^{1}H-NMR spectrum of liquid products collected from the anolyte.

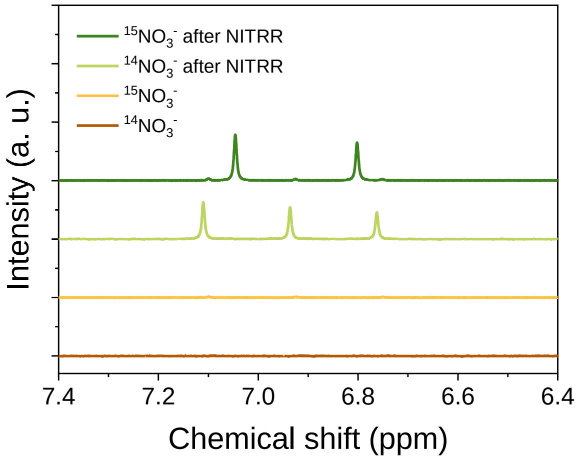

Supplementary Fig. 49. Product analysis. Representative ^{1}H-NMR spectrum NMR spectra before and after NITRR using $^{15}NO_3^-$ and $^{14}NO_3^-$ electrolytes. Without the electrochemical reactions, neither the $^{15}NO_3^-$ and $^{14}NO_3^-$ electrolytes showed NH_4^+ signals. After electrocatalytic NITRR using L-Cu_xO-HC with $^{14}NO_3^-$, three peaks corresponding to $^{14}NH_4^+$ production were observed. When performing NITRR with $^{15}NO_3^-$, the two peaks of $^{15}NH_4^+$ were detected, which confirmed that all our NH_3 products were generated by electrochemical NITRR rather than contaminants.

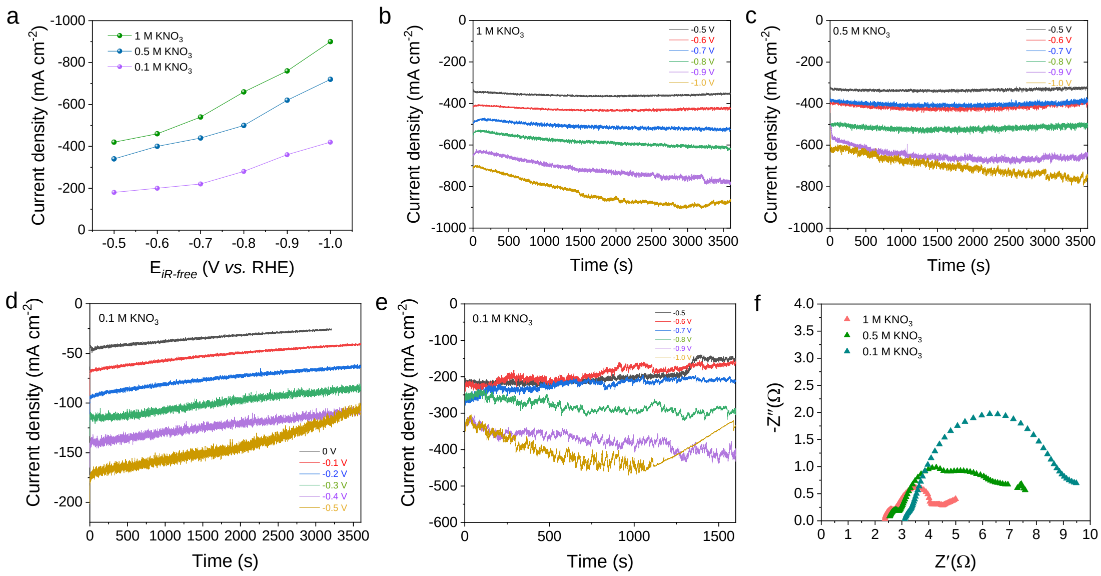

Supplementary Fig. 50. Electrochemical performances. (a) Current density of L-Cu_xO-HC at different potential in different electrolytes. Chronoamperometry in (b) 1 M KOH + 1 M KNO_3, (c) 1 M KOH + 0.5 M KNO_3 and (d, e) 1 M KOH + 0.1 M KNO_3, (f) Nyquist plots in different electrolytes.

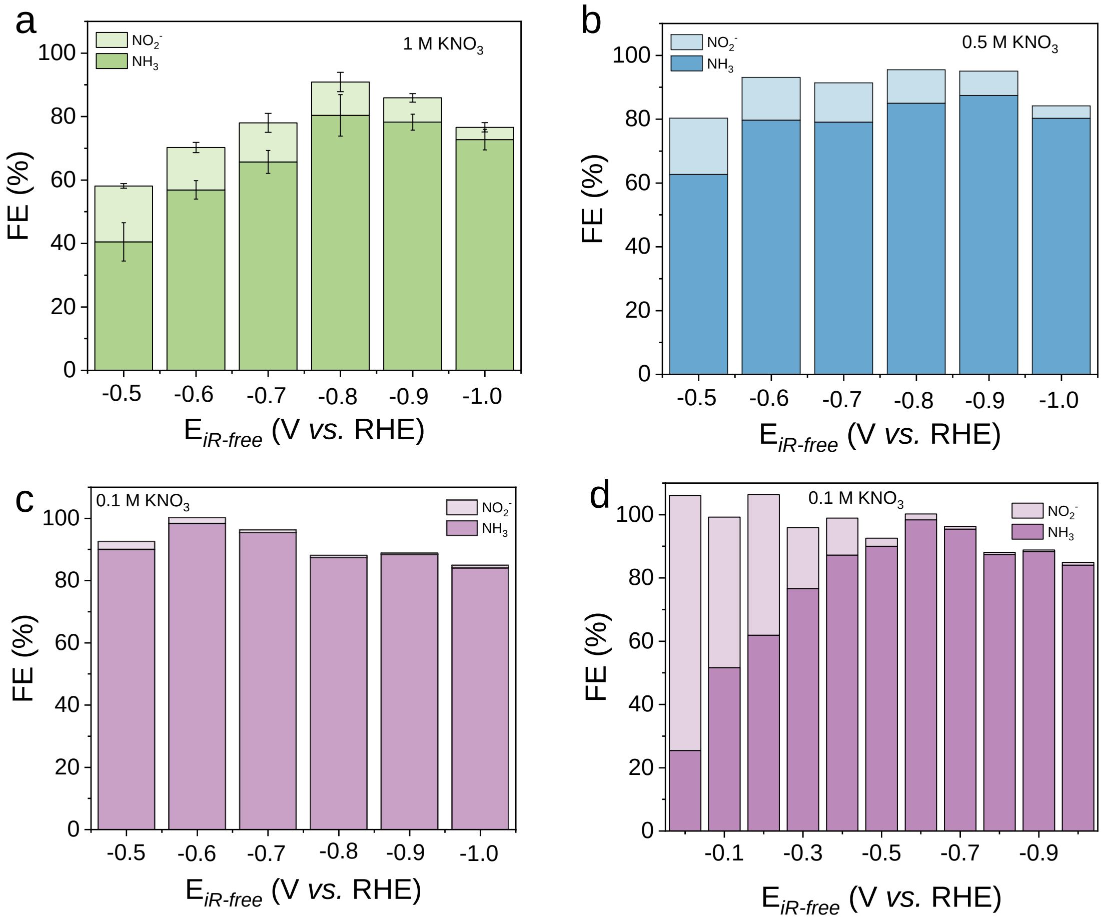

Supplementary Fig. 51. Electrochemical performances. FE of NH_3 and NO_2^- in (a) 1 M KOH + 1 M KNO_3 (b) 1 M KOH + 0.5 M KNO_3 and (c, d) 1 M KOH + 0.1 M KNO_3. The error bars indicate standard deviation among values from three repeated measurements.

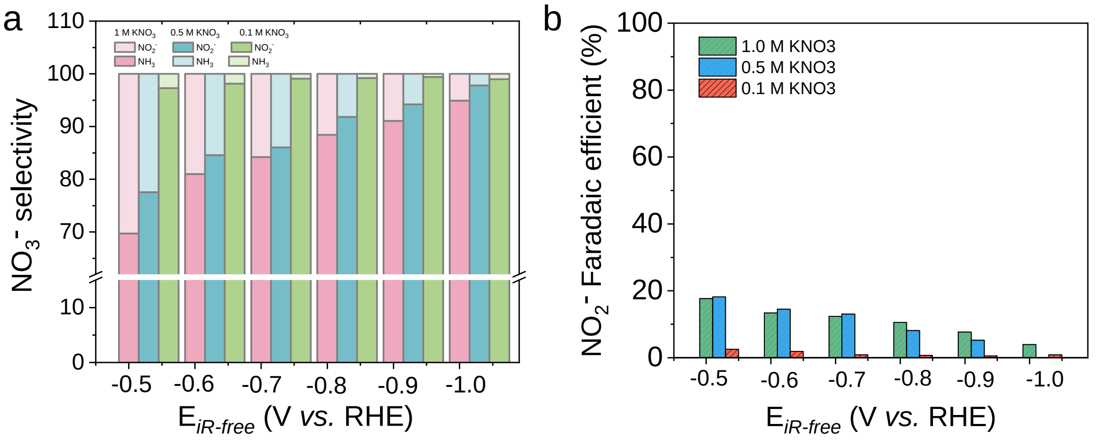

Supplementary Fig. 52. **Electrochemical performances.** (a) Comparison of NO_3^- selectivity and (b) FE of NO_2^- of L-Cu_xO-HC at different potential in in different electrolytes.

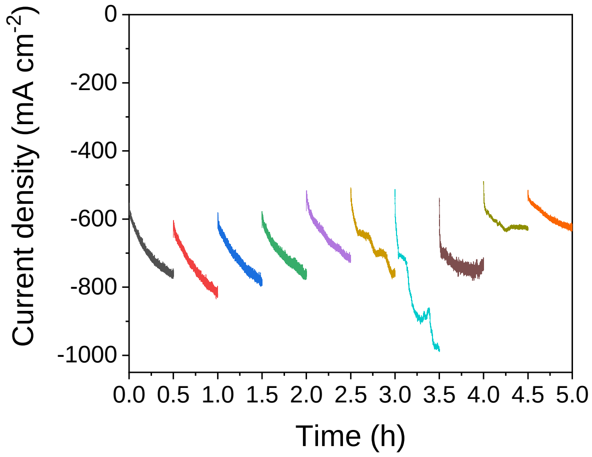

Supplementary Fig. 53. **Electrochemical performances.** Chronoamperometry during the cycles test of L-Cu_xO-HC.

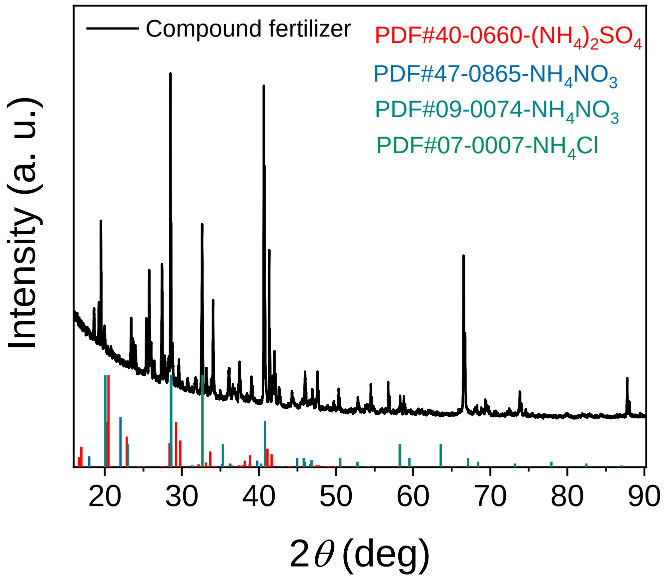

Supplementary Fig. 54. Compound fertilizer XRD analysis result.

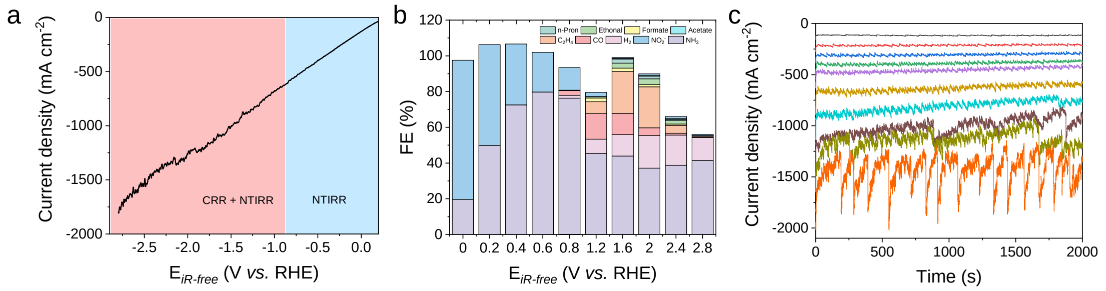

Supplementary Fig. 55. Electrochemical performances. (a) Linear scan voltammetry curves of L-Cu$_x$O-HC in 1 M KOH + 1 M KNO3 with CO_2 in flow cell. (b) FE of different products and (c) Chronoamperometry at different potential.

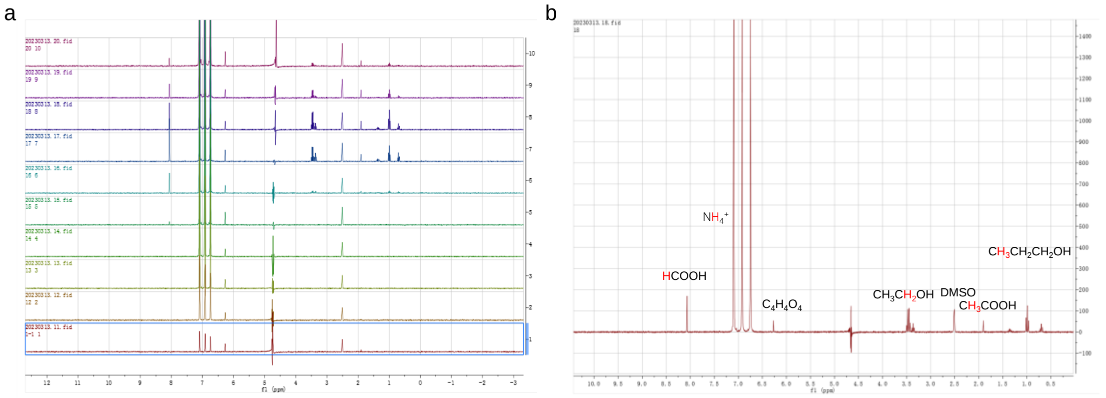

Supplementary Fig. 56. Product analysis. (a) NMR spectra of liquid products of L-Cu$_x$O-HC in 1 M KOH + 1 M KNO3 with CO_2 in flow cell. (b) Representative ^{1}H-NMR spectrum of liquid products enlarges form (a). (NTIRR and CO_2RR-conbined system).

Supplementary Note 1 OH^- absorption test

From a mathematical point of view, this principle of OH^- absorption test is following the interaction energy of an adsorbate under an applied electric field which can be written as follows[5-7] :

$$E_{ads} = E_0 + \mu\varepsilon - \frac{a\varepsilon^2}{2}$$

where E_{ads} represents the adsorption energy at a given strength of electric field; E_0 is the adsorption energy in the absence of electric field; μ and α are the intrinsic dipole moment and polarizability values for the adsorbate which is -0.26 eÅ and 0.14 eÅ^2 V^{-1} for OH^-, respectively[7]; and ε refers to the electric field. It can be seen from the calculation that the stronger the electric field effect, the lower the adsorption energy of OH^-, therefore OH^- could be absorbed at a lower voltage under the influence of stronger electric field.

It is pronounced OH_{ad} peaks associated with Cu (100) facets on L-Cu_xO on the potential of ~0.4 V. Electrochemical adsorption of OH^- on the L-Cu_xO follow this formula[8]: $Cu_xO + OH^- \rightarrow Cu_xO\ (OH)_{ad} + e^-$. When a positive potential is applied, the electrode surface material will adsorb the anion OH^-. Thus, the higher the applied potential, the easier the adsorption of the OH^-. If the OH^- adsorption reaction can occur at a relatively lower potential on a certain material, it means that the material is more likely to adsorb OH^- compared to other materials[8,9].

Supplementary Table 1. The radius of typical alkali metal cations in different states

Cations	R_{ionic} (Å)	$R_{ion\text{-}water}$ (Å)	R_{Stokes} (Å)	$R_{cStokes}$ (Å)
Li^+	0.76	2.08	2.38	3.82
K^+	1.50	2.79	1.25	3.31
Cs^+	1.91	3.13	1.19	3.29

Note: R_{ionic} represents the ionic radius of typical cations. $R_{ion\text{-}water}$ represents mean ion-water internuclear distances. R_{Stokes} and $R_{cStokes}$ represent the Stokes and corrected Stokes radius of hydrated ions, respectively[10].

Supplementary Table 2. EXAFS fitting results of the Cu K edge EXAFS for L-CuxO-HC, b L-CuxO-MC and c L-CuxO-LC, respectively.

Sample	path	CN	σ^2	ΔE_0	R	R-factor
L-Cu_xO-HC	Cu-O	3.6(0.2)	0.0047(0.0007)	3.67(0.95)	1.95(0.01)	0.008
	Cu-Cu1	4.9(0.9)	0.0086(0.0013)	6.79(1.73)	2.93(0.01)	
	Cu-Cu2	3.8(0.9)	0.0086(0.0013)	6.79(1.73)	3.12(0.01)	
L-Cu_xO-MC	Cu-O	3.4(0.2)	0.0049(0.0005)	4.21(0.64)	1.95(0.01)	0.004
	Cu-Cu1	3.8(0.6)	0.0086(0.0010)	6.27(1.37)	2.92(0.01)	
	Cu-Cu2	3.1(0.6)	0.0086(0.0010)	6.27(1.37)	3.11(0.01)	
L-Cu_xO-LC	Cu-O	2.8(0.2)	0.0071(0.0014)	4.14(1.09)	1.92(0.01)	0.007
	Cu-Cu	2.5(1.0)	0.0128(0.0040)	-7.44(1.67)	2.91(0.02)	

Supplementary Table 3. Comparison of the CO_2RR performance of the L-CuxO-HC in this work with the previously reported Cu-based catalysts in flow cell.

Catalysts	Electrolytes	Faradaic efficiency (%)		Partial current density (mA cm^{-2})		Refs
		C_2H_4	C_{2+}	C_2H_4	C_{2+}	
**L-Cu_xO-HC**	1 M KOH	60.1	81.2	497.5	665.87	★ This work
	0.5 M K_2SO_4	56.75	72.80	289.41	397.09	
	0.01 M H_3PO_4 + Saturated K_2SO_4	42.83	56.93	210.77	297.66	
**Cu-PTFE-99 NN**	1 M KOH	45.2	85.4	293.3	529.9	11
**Defect-Site-Rich Cu**	1 M KOH	N.A.	71	N.A.	142	12
**Bioinspired Cu dendrites**	1 M KOH	40	64	N.A.	255	13
**Cu-CuI**	1 M KOH	N.A.	71	N.A.	591	14
**Cu/C/PTFE**	1 M KOH	N.A.	~60	N.A.	250	15
**Cu HoMSs**	0.5 M $KHCO_3$	N.A.	77	N.A.	513.7	16
**PIL-Cu(I)**	1 M KOH	N.A.	76.1	N.A.	304.2	17
**$Cu_2P_2O_7$**	1 M KOH	N.A.	73.6	N.A.	350	18
**Cu_3N_x-50-µA**	1 M KOH	N.A.	81.7	N.A.	309	19

Supplementary Table 4. Comparison of the NTIRR performance of the L-CuxO-HC in this work with the previously reported Cu-based catalysts.

Catalysts	Electrolyte	Ammonia Current density (mA cm^{-2})	Ammonia Yield		Ref
			mg h^{-1} mg_{cat}^{-1}	mmol h^{-1} mg_{cat}^{-1}	
**L-Cu_xO-HC**	1 M NO_3^-<br>1 M KOH	654.4	81.83 mg h^{-1} mg_{cat}^{-1}<br>49.09 mg h^{-1} cm^{-2}	4.81 mmol h^{-1} mg_{cat}^{-1}<br>2.88 mmol h^{-1} cm^{-2}	★**This work**
	0.5 M NO_3^-<br>1 M KOH	573	49.81 mg h^{-1} mg_{cat}^{-1}<br>29.89 mg h^{-1} cm^{-2}	2.93 mmol h^{-1} mg_{cat}^{-1}<br>1.758 mmol h^{-1} cm^{-2}	
	0.1 M NO_3^-<br>1 M KOH	353	32.68 mg h^{-1} mg_{cat}^{-1}<br>19.61 mg h^{-1} cm^{-2}	1.92 mmol h^{-1} mg_{cat}^{-1}<br>1.152 mmol h^{-1} cm^{-2}	
**Cu/Cu_2O nanowires**	200 ppm NO_3^-<br>0.5 M Na_2SO_4	75	4.16 mg h^{-1} cm^{-2}	0.2449 mmol h^{-1} cm^{-2}	20
**Cu cylinder**	30 mM KNO_3 in 0.1 M K_2SO_4	60	3.56 mg h^{-1} cm^{-2}	N.A.	21
**Cu-cis-N_2O_2**	1000 ppm KNO_3<br>0.5 M Na_2SO_4	323.76	28.73 mg h^{-1} cm^{-2}	N.A.	22
**$Cu_{70}Zn_{30}$**	0.3 M $NaNO_3$<br>1 M $(NH4)_2SO_4$	600	6.53 mg h^{-1} cm^{-2}	N.A.	23
**$Cu_{70}Ni_{30}$**	0.1 M $NaNO_3$<br>1 M NaOH	400	5.9 mg h^{-1} cm^{-2}	N.A.	24
**Ce-doped Cu**	1400 ppm NO_3^-<br>1 M KOH	242	16.83 mg h^{-1} cm^{-2}	0.99 mmol h^{-1} cm^{-2}	25
**Ru nanoclusters**	1M NO_3^-<br>1 M KOH	251	N.A.	5.56 mmol h^{-1} mg_{cat}^{-1}<br>1.17 mmol h^{-1} cm^{-2}	26
**Cu–PTCDA**	36 mM NO_3^-	40	0.44 mg h^{-1} cm^{-2}	0.0256 mmol h^{-1}	27

	0.1 mM PBS			cm^{-2}	
**polycrystalline Cu**	0.1 M KNO_3 0.5 M Na2SO4	90	1.72 mg h^{-1} cm^{-2}	0.1014 mmol h^{-1} cm^{-2}	28
**Fe/Ni_2P**	500 mM NO_3^- 0.1 M K_2SO_4	170	4.17 mg h^{-1} cm^{-2}	N.A.	29
**ZIFNC@GDY**	$NaNO_3$ Na_2SO_4	230	6.8 mg h^{-1} cm^{-2}	0.40 mmol h^{-1} cm^{-2}	30
**$Cu_{50}Ni_{50}$ alloy**	100 mM NO_3^- 1 M KOH	300	N.A.	N.A.	31
**$Cu_{50}Co_{50}$**	100 mM NO_3^- 1 M KOH	1098	N.A.	4.58 mmol h^{-1} cm^{-2}	32
**Cu-NSs**	10 mM KNO_3 0.1 M KOH	30	0.3901 mg h^{-1} mg_{cat}^{-1}	N.A.	33
**Rh@Cu**	100 mM NO_3^- 0.1 M Na_2SO_4	162	N.A.	1.27 mmol h^{-1} cm^{-2}	34
**FOSP-Cu**	0.1 M KNO_3 0.5 M Na_2SO_4	N.A.	N.A.	101.4 μmol cm^{-2} h^{-1}	28
**Pd Octahedron**	0.1 M NO_3^- 0.1 M Na_2SO_4	N.A.	2.74 mmol h^{-1} mg^{-1}	0.5485 mmol h^{-1} cm^{-2}	35
**MP-Cu**	50 mM NO_3^- 1 M KOH	138	N.A.	0.543 mmol h^{-1} cm^{-2}	36
**CuPd nanocube**	1 M NO_3^- 1 M KOH	310	N.A.	6.25 mmol h^{-1} mg^{-1}	37
**Cu-NBs-100**	0.1 M NO_3^- 1 M KOH	280	N.A.	0.650 mmol h^{-1} mg^{-1}	38

Supplementary References

1 Ringe, S. *et al.* Understanding cation effects in electrochemical CO_2 reduction. *Energy Environ. Sci.* **12**, 3001-3014, (2019).

2 Gu, J. *et al.* Modulating electric field distribution by alkali cations for CO_2 electroreduction in strongly acidic medium. *Nat. Catal.* **5**, 268–276, (2022).

3 Endrodi, B. *et al.* Operando cathode activation with alkali metal cations for high current density operation of water-fed zero-gap carbon dioxide electrolyzers. *Nat. Energy.* **6**, 439-448, (2021).

4 Yu, J. *et al.* Interfacial electric field effect on electrochemical carbon dioxide reduction reaction. *Chem Catal.* **2**, 2229-2252, (2022).

5 Che, F. *et al.* Elucidating the roles of electric fields in catalysis: A perspective. *ACS Catal.* **8**, 5153-5174, (2018).

6 Chen, L. D., Urushihara, M., Chan, K. & Nørskov, J. K. Electric field effects in electrochemical CO_2 reduction. *ACS Catal.* **6**, 7133-7139, (2016).

7 Resasco, J. *et al.* Promoter effects of alkali metal cations on the electrochemical reduction of carbon dioxide. *J. Am. Chem. Soc.* **139**, 11277-11287, (2017).

8 Cao, Y. *et al.* Surface hydroxide promotes CO_2 electrolysis to ethylene in acidic conditions. *Nat. Commun.* **14**, 2387, (2023).

9 Dinh, C. T. *et al.* CO_2 electroreduction to ethylene via hydroxide-mediated copper catalysis at an abrupt interface. *Science.* **360**, 783-787, (2018).

10 E. R. Nightingale, J. Phenomenological theory of ion solvation. effective radii of hydrated ions. *J. Phys. Chem. B.* **63**, 1381-1387, (1959).

11 Yang, B. *et al.* Accelerating CO_2 electroreduction to multicarbon products via synergistic electric–thermal field on copper nanoneedles. *J. Am. Chem. Soc.* **144**, 3039−3049, (2022).

12 Gu, Z. *et al.* Efficient electrocatalytic CO_2 reduction to C_{2+} alcohols at defect-site-rich cu surface. *Joule.* **5**, 429-440, (2021).

13 Niu, Z. Z. *et al.* Hierarchical copper with inherent hydrophobicity mitigates electrode flooding for high-rate co2 electroreduction to multicarbon products. *J. Am. Chem. Soc.* **143**, 8011-8021, (2021).

14 Li, H. *et al.* High-rate CO_2 electroreduction to C_{2+} products over a copper-copper iodide catalyst. *Angew. Chem., Int. Ed. Engl.* **60**, 14329-14333, (2021).

15 Xing, Z. *et al.* Enhancing carbon dioxide gas-diffusion electrolysis by creating a hydrophobic catalyst microenvironment. *Nat. Commun.* **12**, 136, (2021).

16 Liu, C. *et al.* Nanoconfinement engineering over hollow multi-shell structured copper towards efficient electrocatalytical C-C coupling. *Angew. Chem., Int. Ed. Engl.* **60**, e202113498, (2021).

17 Xu, B. H. *et al.* Highly efficient electrocatalytic CO_2 reduction to C_{2+} products on a poly(ionic liquid)-based Cu(0)-Cu(I) tandem catalyst. *Angew. Chem., Int. Ed. Engl.* **61**, e2021106, (2022).

18 Sang, J. *et al.* A reconstructed $Cu_2P_2O_7$ catalyst for selective CO_2 electroreduction to multicarbon products. *Angew. Chem., Int. Ed. Engl.* **61**, e202114238, (2021).

19 Peng, C. *et al.* Lithiation-enabled high-density nitrogen vacancies electrocatalyze CO_2 to C_2 products. *Adv. Mater.* **33**, 2103150, (2021).

20 Wang, Y. *et al.* Unveiling the activity origin of a copper-based electrocatalyst for selective nitrate reduction to ammonia. *Angew. Chem., Int. Ed. Engl.* **59**, 5350-5354, (2020).

21 González Pérez, O. & Bisang, J. M. Removal of nitrate using an activated rotating cylinder electrode. *Electrochim. Acta.* **194**, 448-453, (2016).

22 Xue-Feng Cheng *et al.* Coordination Symmetry Breaking of Single Atom Catalysts for Robust and Efficient Nitrate Electroreduction to Ammonia. *Adv. Mater.* **34**, 2205767, (2022).

23 Mattarozzi, L. *et al.* Electrodeposition of compact and porous Cu-Zn alloy electrodes and their use in the cathodic reduction of nitrate. *J. Electrochem. Soc.* **162**, D236-D241, (2015).

24 Mattarozzi, L. *et al.* Hydrogen evolution assisted electrodeposition of porous Cu-Ni alloy electrodes and their use for nitrate reduction in alkali. *Electrochim. Acta.* **140**, 337-344, (2014).

25 Yang, L. *et al.* Interface engineering cerium-doped copper nanocrystal for efficient electrochemical nitrate-to-ammonia production. *Electrochim. Acta.* **411**, 140095, (2022).

26 Li, J. *et al.* Efficient ammonia electrosynthesis from nitrate on strained ruthenium nanoclusters. *J. Am. Chem. Soc.* **142**, 7036-7046, (2020).

27 Chen, G.-F. *et al.* Electrochemical reduction of nitrate to ammonia via direct eight-electron transfer using a copper–molecular solid catalyst. *Nat. Energy.* **5**, 605-613, (2020).

28 Zhao, Y. *et al.* Flower-like open-structured polycrystalline copper with synergistic multi-crystal plane for efficient electrocatalytic reduction of nitrate to ammonia. *Nano Energy.* **97**, 107124, (2022).

29 Zhang, R. *et al.* Efficient ammonia electrosynthesis and energy conversion through a Zn‐nitrate battery by iron doping engineered nickel phosphide catalyst. *Adv. Energy Mater.* **12**, 2103872, (2022).

30 Zhao, S. *et al.* Controlled growth of donor-bridge-acceptor interface for high-performance ammonia production. *Small.* **18**, e2107136, (2022).

31 Wang, Y. *et al.* Enhanced nitrate-to-ammonia activity on copper-nickel alloys via tuning of intermediate adsorption. *J. Am. Chem. Soc.* **142**, 5702-5708, (2020).

32 Fang, J. Y. *et al.* Ampere-level current density ammonia electrochemical synthesis using CuCo nanosheets simulating nitrite reductase bifunctional nature. *Nat. Commun.* **13**, 7899, (2022).

33 Fu, X. *et al.* Alternative route for electrochemical ammonia synthesis by reduction of nitrate on copper nanosheets. *Appl. Mater. Today.* **19**, 100620, (2020).

34 Huimin Liu *et al.* Efficient electrochemical nitrate reduction to ammonia with copper-supported rhodium cluster and single-atom catalysts. *Angew. Chem. Int. Ed. .* **61**, e202202556, (2022).

35 Han, Y. *et al.* Facet-controlled palladium nanocrystalline for enhanced nitrate reduction towards ammonia. *J. Colloid Interface Sci.* **600**, 620-628, (2021).

36 Wen, W. *et al.* Metastable phase Cu with optimized local electronic state for efficient electrocatalytic production of ammonia from nitrate. *Adv. Funct. Mater.* **33**, 2212236, (2022).

37 Gao, Q. *et al.* Breaking adsorption-energy scaling limitations of electrocatalytic nitrate reduction on intermetallic CuPd nanocubes by machine-learned insights. *Nat. Commun.* **13**, 2338, (2022).

38 Hu, Q. *et al.* Reaction intermediates-mediated electrocatalysts synthesis favors specified facets and defects exposure for efficient nitrate-ammonia conversion. *Energy Environ. Sci.* **14**, 4989, (2021).
