# Supplementary material for: Accelerating multielectron reduction at CuxO nanograins interfaces with controlled local electric field
Source: Nat Commun. 2023 Nov 15;14:7383. doi: 10.1038/s41467-023-43303-1 (PMC10651938; doi:10.1038/s41467-023-43303-1)
Supplement: Supplementary file 3 — Description of Additional Supplementary Files [file 41467_2023_43303_MOESM3_ESM.pdf]

Description of Additional Supplementary Files

Supplementary Movie S1. A movie illustrating the formation of oxide nanoparticles by laser irradiation in water. The color changes indicate the transition, including the formation of Cu_xO nanoparticles in 10 min, loosely interconnected agglomerates in 30 min, and bipyramids in 60 min.
